# Properties of Plant Extracts from Adriatic Maritime Zone for Innovative Food and Packaging Applications: Insights into Bioactive Profiles, Protective Effects, Antioxidant Potentials and Antimicrobial Activity

**DOI:** 10.3390/antiox14080906

**Published:** 2025-07-24

**Authors:** Petra Babić, Tea Sokač Cvetnić, Iva Čanak, Mia Dujmović, Mojca Čakić Semenčić, Filip Šupljika, Zoja Vranješ, Frédéric Debeaufort, Nasreddine Benbettaieb, Emilie Descours, Mia Kurek

**Affiliations:** 1 Laboratory for Food Packaging, University of Zagreb Faculty of Food Technology and Biotechnology, Pierottijeva 6, 10000 Zagreb, Croatia; ppisonic@pbf.hr (P.B.); tsokac@pbf.hr (T.S.C.); 2 Laboratory for General Microbiology and Food Microbiology, University of Zagreb Faculty of Food Technology and Biotechnology, Pierottijeva 6, 10000 Zagreb, Croatia; icanak@pbf.hr; 3 Faculty of Agriculture, University of Zagreb, Svetošimunska cesta 25, 10000 Zagreb, Croatia; mdujmovic@agr.hr; 4 Laboratory for Physical Chemistry and Corrosion, University of Zagreb Faculty of Food Technology and Biotechnology, Pierottijeva 6, 10000 Zagreb, Croatia; mcakic@pbf.hr (M.Č.S.); fsupljika@pbf.hr (F.Š.); zvranjes@pbf.hr (Z.V.); 5 BioEngineering Department, IUT-Dijon, University Burgundy Europe, 7 Blvd. Docteur Petitjean, BP17867, 21078 Dijon, CEDEX, France; frederic.debeaufort@ube.fr (F.D.); nasreddine.benbettaieb@ube.fr (N.B.); 6 PCAV Team, Joint Unit UMR PAM, Food Processing and Microbiology, INRAé, Institut AgroDijon, University Burgundy Europe, 1 Esplanade Erasme, 21000 Dijon, France; 7 Institut Supérieur International Parfum Cosmétiques Arômes (ISIPCA), 34-36 rue du parc de Clagny, 78000 Versailles, France; edescours@isipca-lafabrique.fr

**Keywords:** microwave-assisted extraction, phenolic compounds, volatile compounds

## Abstract

Knowledge about the composition (volatile and non-volatile) and functionality of natural extracts from Mediterranean plants serves as a basis for their further application. In this study, five selected plants were used for the extraction of plant metabolites. Leaves and flowers of *Critmum maritimum*, *Rosmarinus officinalis*, *Olea europea*, *Phylliera latifolia* and *Mellisa officinalis* were collected, and a total of 12 extracts were prepared. Extractions were performed under microwave-assisted conditions, with two solvent types: water (W) and a hydroalcoholic (ethanolic) solution (HA). Detailed extract analysis was conducted. Phenolics were analyzed by detecting individual bioactive compounds using high-performance liquid chromatography and by calculating total phenolic and total flavonoid content through spectrophotometric analysis. Higher concentrations of total phenolics and total flavonoids were obtained in the hydroalcoholic extracts, with the significantly highest total phenolic and flavonoid values in the rosemary hydroalcoholic extract (3321.21 mg_GAE_/L) and sea fennel flower extract (1794.63 mg_QE_/L), respectively; and the lowest phenolics in the water extract of olive leaves (204.55 mg_GAE_/L) and flavonoids in the water extracts of sea fennel leaves, rosemary, olive and mock privet (around 100 mg_QE_/L). Volatile organic compounds (VOC) were detected using HS-SPME/GC–MS (Headspace Solid-Phase Microextraction coupled with Gas Chromatography-Mass Spectrometry), and antioxidant capacity was estimated using DPPH (2,2-diphenyl-1-picrylhydrazyl assay) and FRAP (Ferric Reducing Antioxidant Power) methods. HS-SPME/GC–MS analysis of samples revealed that sea fennel had more versatile profile, with the presence of 66 and 36 VOCs in W and HA sea fennel leaf extracts, 52 and 25 in W and HA sea fennel flower extracts, 57 in rosemary W and 40 in HA, 20 in olive leaf W and 9 in HA, 27 in W mock privet and 11 in HA, and 35 in lemon balm W and 10 in HA extract. The lowest values of chlorophyll a were observed in sea fennel leaves (2.52 mg/L) and rosemary (2.21 mg/L), and chlorophyll b was lowest in sea fennel leaf and flower (2.47 and 2.25 mg/L, respectively), while the highest was determined in olive (6.62 mg/L). Highest values for antioxidant activity, determined via the FRAP method, were obtained in the HA plant extracts (up to 11,216 mg_AAE_/L for lemon balm), excluding the sea fennel leaf (2758 mg_AAE_/L) and rosemary (2616 mg_AAE_/L). Considering the application of these plants for fresh fish preservation, antimicrobial activity of water extracts was assessed against *Vibrio fischeri* JCM 18803, *Vibrio alginolyticus* 3050, *Aeromonas hydrophila* JCM 1027, *Moraxella lacunata* JCM 20914 and *Yersinia ruckeri* JCM 15110. No activity was observed against *Y. ruckeri* and *P. aeruginosa*, while the sea fennel leaf showed inhibition against *V. fisheri* (inhibition zone of 24 mm); sea fennel flower was active against *M. lacunata* (inhibition zone of 14.5 mm) and *A. hydrophila* (inhibition zone of 20 mm); and rosemary and lemon balm showed inhibition only against *V. fisheri* (inhibition zone from 18 to 30 mm). This study supports the preparation of natural extracts from Mediterranean plants using green technology, resulting in extracts rich in polyphenolics with strong antioxidant potential, but with no clear significant antimicrobial efficiency at the tested concentrations.

## 1. Introduction

The agricultural and food industries produce massive amounts of waste, spanning both edible and inedible parts from various crops during cultivation and processing. This waste can lead to environmental issues, management difficulties, and economic concerns worldwide [1]. To address these challenges, numerous strategies have emerged aimed at utilizing agricultural and industrial waste to create high-value products. By recovering these residues, their antioxidant and pharmacological benefits can be further explored and accordingly exploited for different purposes.

Plants are capable of producing a diverse array of bioactive compounds found in abundance, and the concentration of bioactive metabolites in spices, herbs or essential oils varies significantly across species and cultivar differences, as well as according to different parts of the plant used, such as differences in roots, seeds, flowers, and leaves [2,3,4,5,6]. Their antioxidant properties are mostly attributed to a variety of compounds, including flavonoids, phenolic acids, tannins and stilbenes. The chemical composition of plants and their derivatives is directly influenced by the edaphoclimatic conditions to which the plants are subjected [2,3,4,5,6]. Recovering bioactive compounds from food-derived waste has emerged as a pivotal area of research within the realm of food science, offering a sustainable and economically viable strategy for the food industry [7], including the food packaging sector. Consequently, there is growing interest in exploring various plant extracts as viable natural antioxidants for active packaging solutions, with the possibility of enhancing the oxidative stability of food products [8,9]. Researchers are also seeking to find different and new natural sources of bioactive compounds. The Adriatic coast counts on a rich variety of species that are indigenous to the Mediterranean Basin, including a wide selection of edible and non-edible plants and aromatic herbs [4,10].

In addition, plastics, as the most often used material for food packaging, contribute to huge amounts of waste and environmental pollution. These problems have led to a growing interest and development of the environmentally friendly packaging based on natural polymers, such as edible films and coatings [11,12] or biobased and/or biodegradable active packaging [13]. Sea fennel (*Crithmum maritimum*), a halophyte plant traditionally found in coastal regions, has recently attracted increasing attention for its nutritional and bioactive properties, as a rich source of hydrophilic (polyphenols and vitamin C) and lipophilic (carotenoids, essential oils and fatty acids) bioactive compounds [14,15,16,17,18,19]. Belonging to a plant group known as halophytes, it has developed a range of adaptations to cope with this stress, which often results in an increased production of beneficial phytochemicals, also known as plant secondary metabolites [4]. As sea fennel is one of the most widespread Mediterranean halophytes and an emerging crop nowadays, its implementation in all steps throughout the food product lifecycle is unavoidable. Therefore, it is not surprising that there is enormous scientific interest in characterizing this unique plant to answer the food and packaging industry demand for more natural and sustainable systems.

Among the various Mediterranean herbs, rosemary (*Rosmarinus officinalis*) is a prominent evergreen shrub in the Lamiaceae family, with a rich profile of bioactive metabolites that contribute to its health-promoting effects, particularly its strong antioxidant capacity, and it also holds significant economic value as an agricultural crop [20]. Despite extensive study of various rosemary extracts, by-products from this unique plant-based industry remain underutilized [21].

The olive tree, a cultivar belonging to the Oleaceae family is currently cultivated worldwide, with higher incidence in the Mediterranean Basin [22]. The characteristics of *O. europaea* L. are shaped by various elements, including soil quality, olive variety and postharvest processing methods. It produces many by-products (leaves, branches, solid and liquid waste); however, olive tree leaves stand out as the part of the tree containing the highest content of phenols, which is often recovered for further functional uses and converted into value-added products [23,24,25].

*Phillyrea latifolia* L., commonly known as mock privet, a plant related to the olive tree and from the Oleaceae family, grows in the Mediterranean region as a type of shrub [26] with economic significance in medicinal and traditional applications, but its by-products are rarely used in the food packaging industry [27]. Even though both the leaf and fruit of mock privet are used for their health-promoting activities, such as antioxidant activity [28], to our knowledge, no studies have compared the profile of volatiles and phenolic compounds in water and hydroalcoholic extracts, related to other antioxidant and antimicrobial properties.

Lemon balm (*Melissa officinalis*) is a medicinal herb with broad therapeutic potential attributed to rich phytochemical profile (volatile compounds, phenolic acids and flavonoids) [29,30].

In order to recover bioactive molecules from plants and prepare plant extracts, choosing an appropriate extraction solvent and extraction method is crucial [31,32]. However, for food applications, safety and environmental concerns must not be neglected. Considering the principles of green chemistry, water is a non-toxic, widely available and environmentally friendly extraction solvent, and due to its polarity, it can effectively extract a wide range of polar bioactive compounds, like phenols [33]. Methanol, ethanol and acetone are also used as extraction solvents, but safety and environmental concerns should be considered [34]. Conventional extraction methods can have limitations regarding efficiency, time, or environmental impact. In contrast, newer eco-friendly extraction technologies have been developed to overcome these challenges, with microwave-assisted extraction (MAE) proving to be among the most suitable ones [31,35]. For example, Bratinčević et al. [31] showed that the highest yield of extracted phenolic compounds was achieved with water/ethanol mixture at a ratio of 1:1 (*v*/*v*). The impact of the solvent nature on extraction efficiency has also been highlighted in studies by Boli et al. [24] and Sánchez-Gutiérrez et al. [36] for olive leaf; by Irakli et al. [37] and Hashem Hashempur et al. [38] for rosemary leaves; Hamieau et al. [39] for lemon balm. To our knowledge, no data are available for mock privet.

Even though many studies are available in the scientific literature on the extraction processes, properties and activities of various plant extracts, there are not many studies focused on a detailed evaluation of the composition and profile of volatiles in extracts prepared via MAE of various plants from the Mediterranean basin, all simultaneously characterised and compared. The selection of the five plants was conducted according to the cutting-edge scientific interest: *Critmum maritimum* (sea fennel) flower and leaf as an Adriatic halophyte; *Rosmarinus officinalis* (rosemary) and *Melissa officinalis* (lemon balm) having a long history of a wide array Mediterranean aromatic herbs with medicine uses; and *Olea europea* (olive) and *Phyliera latifolia* (mock privet) leaves, both belonging to Oleaceae family, the former generally considered agricultural waste after olive harvesting, and the latter a common but still underexploited evergreen tree. A comparison of their composition, antioxidant profiles and antimicrobial activity against five specific fish pathogens (*Vibrio fischeri* JCM 18803, *Vibrio alginolyticus* 3050, *Aeromonas hydrophila* JCM 1027, *Moraxella lacunata* JCM 20914 and *Yersinia ruckeri* JCM 15110) is lacking in the scientific literature. Among comparative studies and analytical approaches available in the literature, plants are characterised using GC–MS analysis of essential oils or dry plants, while in this study, the HS-SPME/GC–MS principle was used for the evaluation of VOCs. The main objective of this study was to assess and compare the phytochemical composition (polyphenol content, total flavonoid content, total chlorophylls and carotenoids, profile of volatiles and non-volatiles), antioxidant (DPPH and FRAP methods) and antimicrobial activity (against selected fish pathogens) of water and hydroalcoholic extracts of selected plant species from the Adriatic area. Therefore, this study provides a detailed analysis of Mediterranean plants, with some of them still highly underexploited. It covers both analysis of volatile and non-volatile fractions of several plants using the same analytical procedures across all of them, enabling detailed comparison of the obtained data. Knowledge on the profile of bioactives in both hydroethanolic and water extracts from chosen plants, obtained via non-invasive and eco-friendly techniques, is of high importance for further applications. In addition, this article provides knowledge about the activity of natural extracts against fish spoilage microorganisms, not commonly studied in the scientific literature but important for their shelf life.

## 2. Materials and Methods

### 2.1. Materials

In this work, the following plant materials were used: sea fennel leaf (*Crithmum maritimum*), olive leaf (*Olea europea*) and mock privet leaf (*Phillyrea latifolia*), all harvested in spring 2024 in Kvarner, Croatia. Sea fennel flower (*Crithmum maritimum*) was harvested in September 2024 in Kvarner, Croatia. Rosemary leaf (*Salvia rosmarinus*) and lemon balm leaf (*Melissa officinalis*) were purchased from Suban Ltd. (Strmec Samoborski, Croatia). Photos of the five plant species used are added in the Supplementary Figure S1. The plant materials were air-dried at 25 °C and 50% relative humidity and stored in a dry place until extraction.

Ethanol, sodium carbonate, sodium acetate trihydrate, potassium acetate and formic acid were purchased from Kemika (Zagreb, Croatia); Folin-Ciocalteu reagent, 2,4,6-tris(2-pycrylhydrazyl) (TPTZ) and 2,2-diphenyl-2-picrylhydrazyl (DPPH) were obtained from Sigma-Aldrich Chemie (St. Louis, MO, USA); hydrochloric acid and aluminum chloride were supplied from Carlo Erba Reagents S.A.S. (Peypin, France); and acetonitrile, acetone and ascorbic acid were purchased from Fisher Chemical (Loughborough, UK). All chemicals used were of analytical reagent grade.

### 2.2. Methods

A schematic diagram outlining the steps involved in this work is presented in Supplementary Figure S2.

#### 2.2.1. Preparation of Extracts—Microwave-Assisted Extraction

Before extraction, the plant material was crushed in the grinder (HR2860/55, Philips, Amsterdam, Netherlands). Water (W) and ethanol/water mixtures in a 1:1 ratio (hydroalcoholic, HA) were used as solvents for the extraction of polar and non-polar compounds, and the mass concentration of the mixture in the solvent was 0.05 g plant/mL. The choice of solvents was made with the intention of using the extracted material in the preparation of food contact materials and food coatings in subsequent research. The extraction was carried out in a round-bottomed flask placed in a microwave reactor (Start S Microwave Labstation for Synthesis, Milestone, Italy). All extractions were conducted under the same conditions: during 10 min at a power of 500 W and at a temperature of 70 °C. After extraction, the samples were filtered through 100% cellulose filter paper to separate the aqueous extract from the solid phase. The extracts were stored at 4 °C until further analysis.

#### 2.2.2. Spectrophotometric Analysis of Total Phenolic and Total Flavonoid Content

The total phenolic content (TPC) was determined spectrophotometrically (UV/VIS spectrometer Lambda 25, Perkin Elmer, Waltham, MA, USA), according to the method described by Shortle et al. [40], which is based on the colorimetric reaction of phenols with the Folin–Ciocalteu (FCu) reagent. A calibration curve was prepared using gallic acid, and the results are given as mg of gallic acid equivalents (GAE) per gram of dry matter in the sample.

The total flavonoid content (TFC) in water and hydroalcoholic extracts was determined using a colorimetric method described by Chang et al. [41]. Shortly, 0.5 mL of extract was mixed with 1.5 mL of 96% ethanol solution, followed by the addition of 100 µL of 10% aluminum chloride, 100 µL of 1 M potassium acetate and 2.8 mL of distilled water. After 30 min of incubation at room temperature, the absorbance of the reaction mixture was measured at 415 nm using the spectrophotometer. Quercetin was used to prepare the calibration curve, and the results are expressed as mg of quercetin equivalents per gram of dry plant material. The measurements were performed in triplicate, and results are presented as mean values ± standard deviation.

#### 2.2.3. HPLC Analysis of Phenolic Compounds

Polyphenolic compounds in freshly prepared extracts were quantified using an HPLC Nexera system (Shimadzu, Kyoto, Japan) coupled with a photodiode and a fluorescence detector. The water extracts were lyophilized in order to remove water for the needs of the experimental setup. The chemical compounds were separated and quantified on a Nucleosil^®^ 100-5 C18 column (5 µm, 250 × 4.6 mm i.d., Macherey-Nagel, GmbH, Düren, Germany). Two mobile phases (A—3% formic acid in HPLC-grade water (*v*/*v*) and B—3% formic acid in HPLC-grade acetonitrile (*v*/*v*)), with gradient elution (A/B: 0–25 min, 90%/10%; 25–30 min, 60%/40%; 30–35 min, 30%/70%; 35–45 min, 90%/10%) were used. The applied sample volume was 20 μL, the mobile phase flow rate was 0.9 mL/min, and the oven temperature was 29 °C. The compounds were analyzed at wavelengths between 220 and 360 nm. For the calibration of selected phenolic compounds, commercial external standards (Sigma Aldrich, Steinheim, Germany) were analyzed under the same conditions. Phenolic compounds were identified by comparing the retention times of the analyzed extracts with the retention times of the standards, and the content of phenolic compounds was calculated from the calibration curves of the standards and expressed as mg/L of plant extract.

#### 2.2.4. Determination of Volatile Compounds via HS-SPME/GC–MS

Volatile compounds were analyzed using headspace solid-phase microextraction coupled with gas chromatography–mass spectrometry (HS-SPME/GC–MS). Approximately 5 g of plant extract was placed into a sealed 10 mL headspace vial. HS-SPME extractions were performed using 1 cm fibers coated with divinylbenzene/carboxen/polydimethylsiloxane (DVB/CAR/PDMS, 50/30 μm), obtained from Supelco (Bellefonte, PA, USA). Prior to analysis, all fibers were conditioned according to the manufacturer’s instructions.

The extraction process was fully automated using an Agilent CTC PAL autosampler (Agilent, Santa Clara, CA, USA) interfaced with the GC–MS system. Samples were pre-equilibrated with stirring for 10 min at 40 °C, followed by extraction for 20 min and thermal desorption of the analytes directly into the GC–MS injector.

Volatile compounds were separated and identified using an Agilent 7890B gas chromatograph coupled to an Agilent 5977A mass spectrometer (Agilent, Santa Clara, CA, USA). Separation was achieved on a DB-5ms capillary column (30 m × 250 μm × 0.25 μm), with helium (≥99.999% purity) as the carrier gas at a constant flow rate of 1.0 mL/min. Injections were carried out in splitless mode at an injector temperature of 260 °C. The GC oven temperature program started at 60 °C (held for 3 min), ramped at 5 °C/min to 240 °C, and was held at this final temperature for 25 min.

The mass spectrometer operated in electron impact (EI) mode at 70 eV with a source temperature of 230 °C. Mass spectra were acquired in full-scan mode over a *m*/*z* range of 30–400. The filament current and quadrupole temperature were set to 150 μA and 250 °C, respectively. Volatile compound identification was carried out by comparing the acquired mass spectra to those contained in the NIST 14 spectral library. A match quality threshold of ≥80% was used for compound acceptance. All measurements were performed in triplicate.

#### 2.2.5. Determination of Total Chlorophylls and Total Carotenoids

Chlorophylls (chlorophyll a and chlorophyll b) and total carotenoids were determined spectrophotometrically by measuring the absorbance of acetone extracts at different wavelengths (662, 644 and 440 nm), according to Holm [42] and Wettstein [43]. The amount of 0.30 ± 0.01 g of fresh plant material was homogenized with a total volume of 15 mL of acetone using a laboratory homogeniser (IKA, UltraTurax T-18, Staufen, Germany). Acetone was added in 3 repetitions. The obtained acetone extracts were filtered through filter paper, and absorbance was measured with a spectrophotometer (UV/VIS spectrometer Lambda 25, Perkin Elmer, Waltham, MA, USA). Measured absorbances were used to quantify pigment compounds using Holm–Wettstein equations [30]:

Chl_a_ = 9.784 × A662 − 0.990 × A644 (mg/L), (1)

Chl_b_ = 21.426 × A644 − 4.65 × A662 (mg/L), (2)

TC_a_ = 4.695 × A440 − 0.268 × TCh (mg/L). (3)

Final contents are expressed as mg/L of plant material. The measurements were performed in triplicate, and results are presented as mean values ± standard deviation.

#### 2.2.6. Determination of Antioxidant Activity Using DPPH and FRAP Method

Antioxidant activity was measured using the 2,2-diphenyl-1-picrylhydrazyl (DPPH) and the ferric-reducing antioxidant power (FRAP) method. The DPPH method involves the reduction of the DPPH radical (prepared at 0.057 mM) in a methanol solution in accordance with the procedure described by Shortle et al. [40]. The ability to scavenge the DPPH radical was expressed as percentage inhibition. The FRAP method involves the reduction of the colorless complex of iron (III) tripyridyltriazine (Fe^3+^-TPTZ) to its ferro form (Fe^2+^), which is an intense blue color, in accordance with the procedure described by Shortle et al. [40]. A calibration curve was prepared using ascorbic acid. The results are expressed as mg equivalent of ascorbic acid per gram of dry plant matter. All measurements were performed in triplicate, and the results are presented as mean values ± standard deviation.

#### 2.2.7. Antimicrobial Activity

The antimicrobial activity was assessed using the agar well diffusion method [44]. *Vibrio fischeri* JCM 18803, *Vibrio alginolyticus* 3050 and *Aeromonas hydrophila* JCM 1027 were cultured in marine broth (Becton Dickinson, Sunnyvale, CA, USA) and incubated aerobically—*Vibrio* strains at 22 °C, and *A. hydrophila* at 37 °C. *Moraxella lacunata* JCM 20914 and *Yersinia ruckeri* JCM 15110 were cultured in nutrient broth (Biolife, Milan, Italy) supplemented with 5 g/L NaCl (Gram-mol, Zagreb, Croatia), while *Pseudomonas aeruginosa* ATCC 27853 was cultured in unsupplemented nutrient broth (Biolife, Milan, Italy). All cultures were incubated aerobically at 37 °C for 24 h. The analysis was performed only on water extracts since high content of ethanol in hydroalcoholic (HA) extracts would definitely impact the activity.

Subsequently, 100 μL of fresh bacterial inoculum was evenly spread on the surface of sterile agar plates prepared with the respective media. Wells were then created in the seeded plates. Two wells were filled with ciprofloxacin (positive control) and distilled water (negative control), respectively. The remaining wells were filled with 30 mg of the lyophilized selected plant extract. The plates were incubated aerobically for 24 h at the appropriate temperature for each bacterial strain. After incubation, the zones of inhibition (ZOI) were measured in millimeters. A larger ZOI indicated bacterial sensitivity to the plant extract, while the absence of a ZOI suggested resistance. All experiments were performed in triplicate.

### 2.3. Statistical Analysis

Statistical analyses (one-way analysis of variance (ANOVA) and Tukey’s multiple comparison tests) were performed using Xlstat-Pro (win) 7.5.3. (Addinsoft, New York, NY, USA). Results were considered significant at *p* > 0.05 confidence level.

## 3. Results and Discussion

Interest in the bioactive potential of plant species for use as natural additives for bioactive packaging to extend or improve food shelf life is growing worldwide. There is limited information about the phytochemical profile, including volatiles from aqueous and hydroalcoholic solutions, and the activity of plant extracts from the north Adriatic coastline, prepared from the wasted plant parts, including information on their antioxidant potential and antimicrobial activity. There is also a lack of comparison of extracts prepared with non-toxic solvents and using non-invasive extraction parameters, ready to be directly used in edible food coatings.

Integration of bioactive extracts into food packaging has attracted increasing attention from the scientific community for several reasons. Increasing interest in sustainable and environmentally friendly food packaging has sparked research into new solutions that address both environmental concerns and consumer health. First of all, extracts can provide additional functionality to produce packaging, as substances offering tailored benefits to enhance food safety, quality or shelf life through their antioxidant and antimicrobial potential. Then, they could extend product shelf life without direct incorporation as food additives, eliminating the need for their consumption. Also, due to the pH sensitivity of certain bioactive compounds, these extracts can act as smart packaging additives, enhancing sensing capabilities effectively. However, most of the activities are in a dose-dependent manner; therefore, knowledge on the composition and the product requirements must not be neglected.

### 3.1. Total Phenolic Content, Total Flavonoid Content and Detailed Chemical Profiles of Plant Extracts

Based on the type of solvent used, the plant materials showed different total phenolic and total flavonoid contents as measured spectrophotometrically (Table 1). Total flavonoids were higher in all HA extracts, regardless of plant type, with the significantly highest values detected in the sea fennel flower extract and the lowest in the water extract of olive leaves and water extract of sea fennel leaves.

The Folin–Ciocalteu (FC) method is commonly used to measure polyphenols in biological materials, but it has some limitations like difficulties in identifying some specific polyphenolic compounds and the risk of interference from sugars and proteins, which can affect the results [45,46]. Therefore, differences between the levels of phenolics measured via chromatography techniques and the levels of TPC obtained using the FC method are expected [47]. Despite these issues, it is useful to compare Folin–Ciocâlteu estimations of total polyphenol levels with chromatographic techniques, such as HPLC and HS-SPME/GC–-MS. Therefore, this approach was followed in this study. Since this spectrophotometric method is quite selective and less sensitive, a detailed analysis of 21 phenolic compounds in freshly prepared extracts was performed via HPLC, and results are given in Table 2. Plants produce about 8000 polyphenols, which are vital for their potent biological properties, including antioxidant, antimicrobial and some other medicinal effects when used as food additives. Their concentrations can vary due to factors like geographical origin, climate, soil salinity and nutrient deficiencies [48,49]. Therefore, the evaluation of raw materials is essential for maximizing the effectiveness of in vitro extracts.

The content of the twenty-one most common organic compounds was analyzed in all samples (Table 2). Compounds were chosen according to the available literature. Of these, 11 were phenolic acids, and 10 were flavonoids. The results are given in Table 2.

According to the literature, various internal and external elements play a crucial role in shaping the quantity, composition and ratios of plant metabolites [47]. Accordingly, different values are noted in the literature, and comparisons are sometimes difficult to provide.

Generally, higher concentrations of phenolics and flavonoids were detected in the hydroalcoholic extracts. A comparison of four compounds, selected due to their high contents detected in the extracts, is given in Figure S3. Chlorogenic acid, rosmarinic acid and naringin were determined as predominant compounds in most extracts, reaching values up to 765, 1282 and 4039 mg/L in sea fennel flower HA, lemon balm HA and sea fennel flower W, respectively. The exception was the Oleaceae family, where oleuropein was predominant (2318 mg/L in mock privet) (Table 2). All other compounds were found in significantly lesser quantities (from traces to <100 mg/L). Among all tested samples, caffeic (6.6 mg/L), coumaric (93.4 mg/L), protocatechuic acid (15.3 mg/L) and myricetin (13.5 mg/L) were detected in the largest proportion in sea fennel flower HA; caffeoylmalic (6.1 mg/L) and ferulic acid (78.9 mg/L) were highest in the sea fennel flower W, while chlorogenic acid, naringin and quercetin-3-glucoside were highest (compared to other plants) in both HA and W of sea fennel flower. 4-Hydroxybenzoic acid (18.2 mg/L) was highest in HA of mock privet, while gallic acid (9.3 mg/L for HA) was found in both mock privet extracts. The highest contents of rosmarinic acid (1282.6 mg/L) and rutin trihydrate (10.3 mg/L) were present in HA of lemon balm; vanilic acid (12.3 mg/L), kaemferol (12.2 mg/L) and quercetin (8.9 mg/L) were highest in HA of rosmeray. Oleuropein was highest in HA olive (2004.9 mg/L) and mock privet (2318.6 mg/L). Carvacrol was detected only in HA of sea fennel flower (10.8 mg/L), olive (13.4 mg/L) and mock privet (30.0 mg/L), while luteolin and apigenin were found in significantly lower quantities, only in traces (up to 2 mg/L).

The polarity of a solvent determines its ability to dissolve substances of different polarity. Since water is highly polar and ethanol is moderately polar [50], the water–ethanol mixture in a ratio of 1:1 (*v*/*v*) used in this study has intermediate polarity. Different water–ethanol mixtures are commonly used to balance polarity and enable the extraction of a broader range of phenolic compounds [51]. Phenolic compounds are generally polar, but the degree of their polarity varies depending on their structure, ranging from slightly to strongly polar [52].

**Figure 1:**
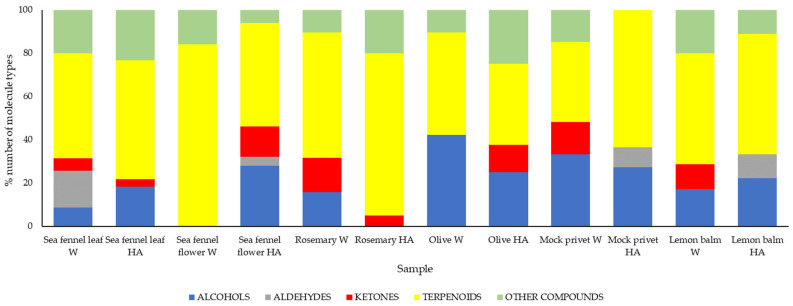
Composition of volatile compounds in 12 selected extracts from 6 plant sources. Percentage distribution of identified molecules according to their chemical families in each sample type (% represents the percentage of compounds by chemical family). Each bar represents the relative proportion in peak surface of compounds belonging to a given chemical family based on the total number of compounds detected in the corresponding extract.

Analysis of extracts via HS-SPME/GC–MS allowed the identification of main volatile compounds. Volatile organic compounds (VOCs) are primarily responsible for plants’ distinct aroma, flavour, and some of their biological activities. The chemical compositions are listed in Tables given in the Supplementary Materials, and they are discussed for each plant separately. The results are generally in line with those reported in the literature, with marked differences in the discussion. Compounds were grouped as alcohols, aldehydes, ketones, terpenoids and other compounds (sesquiterpenoid, alkenes, cycloalkanes, polycyclic aromatic hydrocarbons, organic acids, sulphides, ethers and esters) (Figure 1). A comparison of peak areas of selected compounds that were found in both W and HA extract for each plant is given in Figure S3.

#### 3.1.1. Sea Fennel

Considering the total polyphenol content in sea fennel extracts in the present study, it can be noticed that the content in the HA extract of leaves was higher than in the W extract, whereas the opposite was true for sea fennel flowers. There were no similar results found in the literature. In addition, sea fennel flowers had higher TPC than leaves. The measured values were slightly lower than those given by Veršić Bratinčević et al. [31], but differences are possible due to differences in extraction parameters, notably temperature and time for the extraction, which are indeed known to be crucial for the final extraction efficiency. Additionally, the values are comparable to those reported by Radman et al. [18], who found that TPC in sea fennel methanol extracts obtained via ultrasound extraction was in the range from 3.85 to 26.21 mg_GAE_/g dry plant material, depending on the location of harvest. Nartea et al. [48] showed that TPC of water sea fennel extracts containing both leaves and flowers ranged from 34 to 55 mg_GAE_/g. Furthermore, the same authors analyzed the total flavonoid content in water extracts, with values ranging from 15 to 35 mg_QE_/g, whereas in the present study, this corresponds to 2.446–8.281 mg_QE_/g in water extracts, and 6.670–35.892 mg_QE_/g in HA extracts. Available literature data confirm that it has an excellent source of chlorogenic acid and its isomers, which were also the most abundant compound in HA extracts of flower and leaves (Table 2).

In general, flowers contained higher amounts of hydroxycinnamic acids than leaves, in agreement with previous studies [17,48,53]. Naringin, quercetin-3-glucoside and oleuropein were the predominant flavonoids in both extraction solvents, with significantly higher concentrations in HA.

Similarly, the chlorogenic acid was found to be dominate in methanol–water or ethanol–water extract mixtures of sea fennel, as reported by Radman et al. [18], Popović et al. [4] and Veršić Bratinčević et al. [31]. Also, the authors [18,31] reported that other acids such as ferulic, gallic and protocatechuic acid were present in lower concentrations, similar to the present study.

HS-SPME/GC–MS analysis of the samples revealed the presence of 66 and 36 VOCs in W and HA leaf extract, and 52 and 25 in W and HA flower extract (Tables S1 and S2). The diversity of volatile profiles in sea fennel is responsible for its biological activities. Leaves were dominated by terpenoids, with the highest peak area observed for 4-terpineol (Figure 1, Table S1). 4-Terpineol also had the highest % peak area in the HA extract. When comparing extracts, the peak area in W was 10 times higher than in HA, accounting for a higher number of molecules in W as well. Overall, the principal components are in line with those reported in the scientific literature [54]. Moreover, the VOC profile varied significantly depending on the part of the plant examined, with remarkable variations between leaves and flowers. In flowers, there was significantly higher repartition of terpenoids than other compounds, due to the fact that flowers are rich in essential oils, and detected compounds are usual components [16,49]. In addition, the highest levels of limonene, sabinene and γ-terpinene, as three main compounds in flowers, show similarities to the chemotype characteristic of sea fennel found in Central Italy and wild populations along the Adriatic coastline [16,55,56].

#### 3.1.2. Rosemary

The total polyphenolic content in rosemary extracts was 836 mg_GAE_/L for the water, and >3000 mg_GAE_/L for the HA extract. Values were significantly higher than those given by Škugor Rončević et al. [57], but similar to Nguyen-King et al. [58], Bianchin et al. [59] and Pontillio et al. [60]. Pontillio et al. [60] showed that the highest TPC value was obtained using microwave-assisted extraction with 50% ethanol as the extraction solvent, similar to the present study. The total flavonoid content of the lyophilized ethanol extract in research by Pontillo et al. [60] was 11.89 mg_QE_/g, whereas in the present study, values varied from 12.07 mg_QE_/g for the HA extract to 2.47 mg_QE_/g for the water extract. The biological properties of rosemary have been attributed to its phytochemical composition, mainly rosmarinic and carnosic acid [61,62]. Indeed, rosmarinic acid was the dominant phenolic acid found in both rosemary extracts (61.37 mg/L and 326.55 mg/L in W and HA, respectively), while naringin was dominant among flavonoids (75.76 mg/L and 337.54 mg/L in W and HA, respectively). As it can be noticed, a significantly higher content of rosmarinic acid was quantified in the hydroalcoholic extracts, followed by 4-hydroxybenzoic acid and vanilic acid in the water extract, while in protocatechuic and ferulic acids were in higher in the HA extract. For example, Athanasiadis et al. [63] found that the quantity of rosmarinic acid was dependent on the extraction methods and ethanol–water solvent mixtures. Sharma et al. [64] found 5 log scale differences among seven different extraction methods used, while Al Samer et al. [65] found the influence of the solvent. Authors found that coumaric acid, quercetin and kaempferol were present in higher concentrations in rosemary ethanol extract, with rosmarinic acid at a concentration of 30.90 mg/L, values closer to those of the water extract rather than the hydroalcoholic extracts in the present study.

Volatile profiles of rosemary extracts are given in Table S3. Results confirm that rosemary extracts contain a rich volatile profile, with 57 VOCs found in the water extract and 40 in HA. Both extracts were primarily composed of terpenoids (Figure 1). While compounds such as eucalyptol, α-pinene and camphor have been previously reported as dominant in hydrosol extracts [66], in the present study, there were also camphene, beta-myrcene, d-limonene, p-cymene, camphor and bornyl acetate in higher proportions (Table S3). Similar to Rafya et al. [66], camphor was predominant in the water extract, followed by linalool, 4-terpineol, alpha terpineol, borneol and verbenone. In addition to non-volatile phenolics, detected volatiles also contribute to the characteristic flavour and bioactivity of rosemary. The HA extract had 100 times more alpha pinene, camphene and beta myrcene than the W extract, and 10 times more p-cymene, while W had more 4-terpineol, alpha terpineol, borneol and camphor.

#### 3.1.3. Olive and Mock Privet

The total polyphenol content in the HA olive extract was higher than the content obtained using water as the extraction solvent, while no differences were observed in mock privet samples. In general, hydroalcoholic extracts had a significantly richer profile, with noticeable presence (>10 mg/L) of rosmarinic acid, naringin, carvacrol, kaempferol, ferulic acid and quercetin-3-glycoside. This profile differs from those reported by Šimat et al. [47] and Cukrov et al. [67], who found luteolin and apigenin derivatives as the major flavonoid representatives. Mock privet was richer in ferulic acid, protocatechuic acid, 4-hydroxybenzoic acid, naringin, carvacrol and quercetin-3-glucoside compared to olive samples. The effects of solvents on the olive leaf profile and TPC were previously noticed in the work of Šimat et al. [47] and Debib et al. [68]. In addition, Šimat et al. [47] identified 66 individual phenolic compounds in hydroalcoholic olive leaf extract, belonging to different classes of bioactives and plant metabolites. The same authors also reported differences depending on variety and other factors, with total phenolic content determined via the FC method ranging from 86.73 to 113.60 mg_GAE_/g, values that were higher than those found in the present study. The difference might also be attributed to the presence of oleuropein, which was the predominant compound extracted as detected via HPLC analysis (Table 2). Indeed, it has been previously shown that for the effective extraction of oleuropein from olive leaves, there is the need of a mixture of an organic solvent with water [69], probably due to the lower polarity levels of ethanol compared to water, which reduces the dielectric constant of the solvent and facilitates the solubility and diffusion of desired target compounds (phenolic compounds) in the solvent [70]. Its presence is very important, since oleuropein is the primary molecule responsible for the olive leaf extract characteristics, including its powerful antioxidant and antibacterial activities.

Even though the hydroalcoholic extract of mock privet had a higher content of oleuropein, reflecting a similar trends found in total flavonoid content, there was a difference in total phenolic content. This was possibly due to the presence of some other phenolic compounds, like luteolin-7-O glucoside and 5-caffeoylquinic acid [26], which were not studied in the present work. Similarly, Romero-Diez et al. [71] and Wang et al. [72] observed that the aqueous extract exhibited superior extraction efficiency for phenolic compounds compared to the methanol extract. The results obtained, regardless of the solvent used, reveal that mock privet leaves contain a significant amount of phenolic compounds.

Others found that total polyphenol contents ranged from 10 to 20 mg_GAE_/g DM of olive leaves for HA solution, and 10–15 mg_GAE_/g DM of leaves for water extracts, and 6.29–49.36 mg_GAE_/g dry leaves [73,74], similar to results in the present study. Irakli et al. [37] found around 60 mg mg_GAE_/g DM of plant material in MAE extracts of mock privet, which is higher than in the present study. However, authors used higher proportions of ethanol (70%), which might have influenced extraction efficiency.

The profiles of volatiles in olive and mock privet extracts are given in Tables S4 and S5. This is for the first time that the analysis of VOCs in aqueous and hydroalcoholic extracts of mock privet leaves was revealed using HS-SPME/GC–MS. Results demonstrated significantly lesser diversity of compounds compared to sea fennel and rosemary. In olive leaf extracts, there were 20 VOCs in W and 9 in HA extract, while mock privet had 27 in water and 11 in HA extract. All extracts from the Oleaceae family had principally terpenoids, followed by alcohol. Linalool was predominant in mock privet HA extract, and eucalyptol, camphor and borneol were predominant in the W extract. Other VOCs found in mock privet belong to alcohols and secondary alcohols like 2-octanol and 2-ethyl-1-hexanol, ketones like 2-heptanone and others.

In both olive leaf extracts, alcohols and terpenoids appeared to be dominant, similar to previously reported results [75]. In the water extract, there were principally alcohols and secondary alcohols like 3-methyl-1-butanol, phenyl ethyl alcohol and 2-methyl-1-propanol, responsible for odor of olive leaf volatiles and green, fruity or earthy scent, with notes of herbaceousness and bitterness [76]. Even though linalool was not a primary component of olive leaf extracts, it was detected in the water extract. In mock privet, terpenoids were followed by alcohols in both solvents, with a significant odour impact from eucalyptol and camphor in HA, and from linalool in HA. To the best of our knowledge, no literature data are available for comparison.

#### 3.1.4. Lemon Balm

The total polyphenol content in lemon balm extracts determined in this research was similar for water and HA extracts, with values of 22.54 and 23.98 mg_GAE_/g, respectively. Ordaz et al. [77] reported 5.58–49.19 mg_GAE_/g for water lemon balm extracts prepared via conventional solid–liquid extraction under different extraction conditions of temperature, time and sample quantity. Also, Table 1 shows no significant difference in total flavonoid content between water and HA lemon balm extracts. The obtained values are in accordance with literature data.

The data from the HPLC analysis of the lemon balm extracts reveal that rosmarinic acid was dominant, followed by ferulic acid in notable concentrations in both extracts, while protocatechuic and chlorogenic acids were present at higher concentrations in the hydroalcoholic extract. Considering the flavonoids, naringin and quercetin-3-glucoside were present in the water lemon balm extract, but hydroalcoholic extract was dominated by naringin, quercetin-3-glucoside, kaempferol and rutin. Rutin and aglycones were found in the hydrolysed extract and in very low quantities in the water extract. Similar observations were previously reported by Sentkowska et al. [78]. The high presence of rosmarinic acid in ethanolic lemon balm extracts, followed by rutin, caffeic and protocatechuic acids, has also been previously reported [29,79].

Nurzyńska-Wierdak et al. [80] reported the presence of 106 VOCs in lemon balm leaves, while the total of 35 in the water extract and 10 in the HA extract was found in the present study (Table S6). The predominant constituents of the HA extract were monoterpenes (D-limonene, rose oxide, l-menthol, D-carvone and citronellol), while in W, terpenoids were also dominant (eucalyptol, l-menthol, citronellol, cyclohexanol and 5-methyl-2-(1-methylethyl), (1.alpha.,2.beta.,5.beta.)) but with smaller differences in proportion compared to alcohols (2-ethyl-1-hexanol, 1-octen-3-ol and cis-3-hexanol) and ketones (3-octanone and acetoin).

### 3.2. Total Chlorophylls and Total Carotenoids

**Figure 2:**
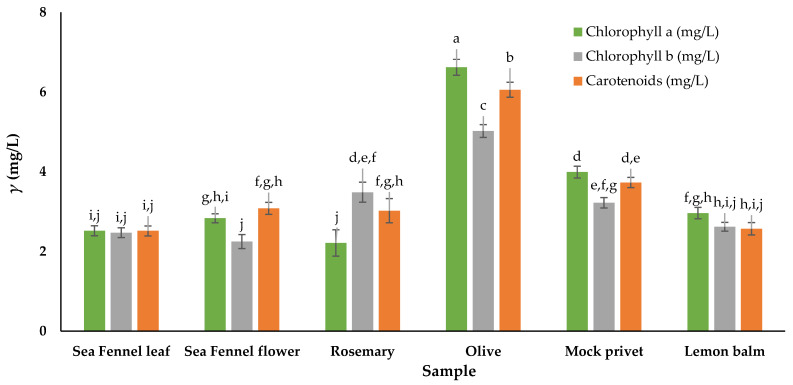
Chlorophyll a, chlorophyll b and total carotenoids in plant material. ^a–j^ Different lowercase superscript letters within a column denote significant differences (*p* > 0.05) between values obtained for different plant materials. Results are given as mean ± standard deviation.

Chlorophylls and carotenoids, the primary pigments found in plants, are valued not only for their colour properties but also for their functional roles, including antioxidant, antimicrobial, anti-cancer, and anti-inflammatory properties. The values of chlorophyll a, chlorophyll b and total carotenoids in all tested samples are given in Figure 2. The lowest value of chlorophyll a was observed in rosemary, and the lowest chlorophyll b content in sea fennel flower, while the highest levels were determined in olive. Flowers had slightly higher mass concentrations of carotenoids than leaves, similar to the findings of Nartea et al. [48], but not significant. Spectrophotometric analysis of total carotenoid reported in the literature show a wide range of content, from a maximum of 470 mg/kg DW in the whole sea fennel plant [81], 338 mg/kg in edible leaves [82], 62.2 mg/kg DW in aerial parts [83], to a minimum of 2.43–4.25 mg/kg DW in leaf tissue of salt-stressed hydroponically grown sea fennel [84]. Additionally, leaves of sea fennel can be considered a good source of lutein, belonging to the carotenoid group. Rosemary had the lowest carotenoid and chlorophyll a content, which agrees with the literature [20,63]. Moreover, literature data report different values for chlorophylls and carotenoids depending on the extraction method and plant variety [63,85] but still comparable to the present study. Olive leaves had significantly more chlorophylls than the other samples. Rosemary had higher chlorophyll b content, which was opposite to Renna et al. [86], who found higher content of chlorophyll a (0.554 mg/g fresh plant material) but lower chlorophyll b (0.181 mg/g fresh plant material). Values for mock privet were hardly comparable, since not many similar studies were available in the scientific literature. In addition, Gori et al. [87] found that the content of the photosynthetic pigments varies not only seasonally but also daily. Irakli et al. [37] found 0.086, 0.061 and 0.085 mg/g dry plant material of chlorophyll a, chlorophyll b and total carotenoids in mock privet, which are lower than values found in the present study. While Dogan et al. [88] found lower content, Šic Žlabur et al. [30] reported significantly higher values of chlorophyll a, chlorophyll b and total carotenoids in lemon balm leaves, with possible differences due to different climate conditions, harvest location and extraction methods [30].

### 3.3. Antioxidant Activity of Plant Extracts Determined Using DPPH and FRAP Method

**Figure 3:**
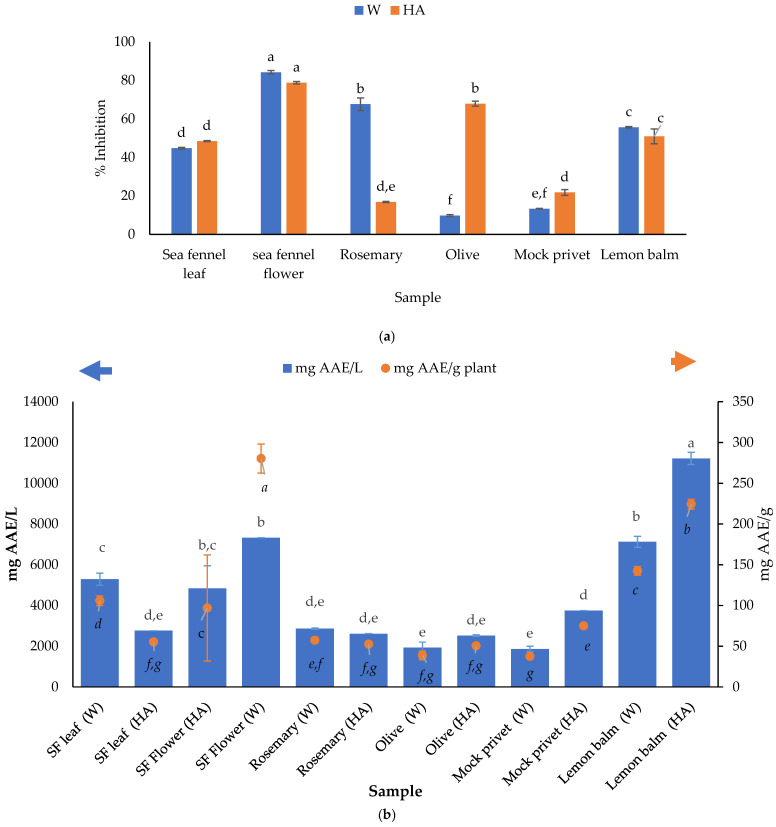
Antioxidant activity of water (W) and hydroalcoholic (HA) extracts determined using (**a**) DPPH method; (**b**) FRAP method. SF—sea fennel. ^a–g^ Different letters denote significant differences (*p* > 0.05) between values obtained for different plant materials. Results are given as mean ± standard deviation, and statistical analysis were performed separately for each series—one for mg AAE/L (given above) and one for mg AAE/g plant (given below).

Two different assays, DPPH and FRAP, were used to evaluate the antioxidant capacity of water and hydroalcoholic plant extracts. The results are presented in Figure 3. Both methods are commonly used to assess antioxidant capacity but differ in their mechanisms and the types of antioxidants they best measure. DPPH measures the ability of antioxidants to scavenge free radicals, while FRAP measures the ability of antioxidants to reduce ferric ions (Fe^3+^) to ferrous ions (Fe^2+^). Antioxidant activity is directly related to the content of antioxidants in the sample, so it is expected that higher content of determined polyphenols results in higher activity. However, it is also possible that some compounds are not detected with applied analytical methods, which might be crucial for the final result. For the most part, polyphenols suppress the generation of free radicals, thus reducing the rate of oxidation by inhibiting the formation of, or deactivating, active species and precursors of free radicals.

In general, higher values for antioxidant activity determined using the FRAP method were obtained in HA plant extracts, excluding the sea fennel leaf and rosemary (Figure 3). In addition, no significant differences in FRAP values were detected between water and HA extracts of sea fennel flower, rosemary and olive leaf. Remarkably high antioxidant activity measured via FRAP was detected in lemon balm, while other extracts showed lower activity. It is quite challenging to directly compare the results of this study (as given in Table 1 and Table 2) with those previously documented. However, in general, the observed antioxidant activity tends to be significantly high. This can be largely attributed to differences in the phenolic profiles of the various leaf extracts and the specific chemical structures of the phenols present in samples.

The obtained values for rosemary and sea fennel in this research were higher than those reported in the literature, while values for lemon balm, olive and mock privet were lower. Examples of results found in the literature are given in Table 3 alongside the results obtained in this study.

To compare, values for FRAP obtained for sea fennel water and hydroalcoholic extracts were significantly higher than those given by Chatzmitakos et al. [89]. The obtained values amounted to 105.77 mg_AAE_/g for sea fennel leaf and 52.82 mg_AAE_/g for sea fennel flower water extracts, and 55.16 and 96.83 mg_AAE_/g dry plant material for the HA extracts. Athanasiadis et al. [63] reported that FRAP values for rosemary ranged from 0.54 to 28.52 mg_AAE_/g, and DPPH values were in the range of 1.00–25.12 mg_AAE_/g, depending on the extraction method. Kontogianni et al. [90] demonstrated that the antioxidant activities of commercial rosemary extracts depend on the concentration of phenolic diterpenes and caffeoyl derivatives (like rosmarinic acid—the most abundant compound found in rosemary extract, Table 2). Lahcene et al. [91] recently found higher values for ethanolic (258.76 mg_GAE_/g extract) than for water (153.720 mg_GAE_/g extract) olive leaf extracts, which were both significantly higher than those found in this study. Lemon balm was the best antioxidant as measured via FRAP, although with slightly lower values than those reported in the literature.

To assess the free radical scavenging capacity of various samples, the DPPH assay is a widely used and reliable method based on the antioxidant scavenging ability of the samples against stable DPPH free radicals. The results for DPPH radical inhibition were similar and remarkably high (from 78.3 to 88.83% inhibition) and did not correlate with TPC.

### 3.4. Antimicrobial Activity

**Figure 4:**
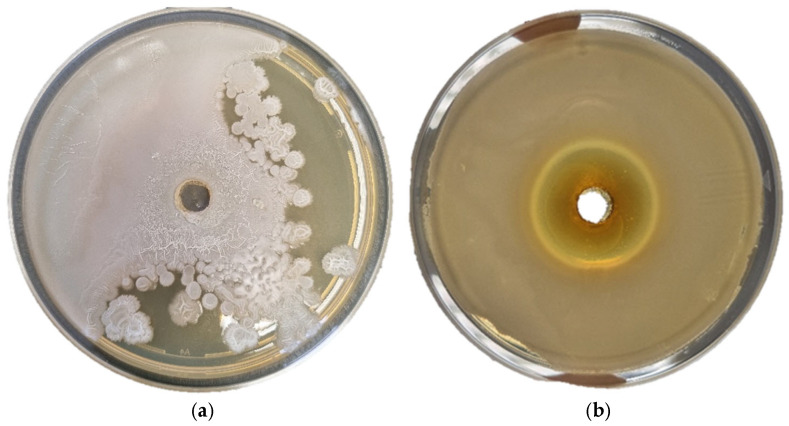
Examples of agar well diffusion method results for mock privet extract: (**a**) no inhibition zone for *Y. ruckeri* and (**b**) good inhibition of *M. lacunata*.

A comparative analysis of the antimicrobial properties of various Mediterranean plant species revealed that extracts from olive (*Olea europaea*) and mock privet (*Phylliera latifolia*) exhibit significantly stronger antimicrobial effects than those from rosemary (*Rosmarinus officinalis*), lemon balm (*Melissa officinalis*) and sea fennel (*Crithmum maritimum*). This difference is primarily attributed to the chemical composition of the plant extracts, particularly the presence of phenolic compounds known for their bioactivity [92]. The inhibition zones of the tested extracts are given in Table 4, with an example of results obtained with inhibition zones shown in Figure 4.

No activity was observed against *Y. ruckeri* and *P. aeruginosa*, while sea fennel leaf showed inhibition against *V. fisheri*, sea fennel flower was active against *M. lacunata* and *A. hydrophila*, and rosemary and lemon balm showed inhibition only against *V. fisheri.* Kraouia et al. [49] showed that essential oil of sea fennel was active against several *Pseudomonas* species. However, the extracts in this study did not show any activity, probably due to insufficient concentrations of active substances in the tested extracts. The largest inhibition zones were observed for members of the Oleaceae family. It was previously shown (Table 2, Tables S4 and S5) that leaves of both olive and mock privet are rich in polyphenols such as oleuropein, and VOCs such as eucalyptol and camphor, known for their antimicrobial activity. In addition to mentioned, in the literature, it was given that, apart from oleuropein and hydroxytyrosol, there is also verbascoside, which exerts bactericidal effects through various mechanisms—including disruption of microbial cell membranes, inhibition of enzymatic systems and reduction of oxidative stress. Numerous studies have confirmed that these compounds possess a broad spectrum of antimicrobial activity, particularly against Gram-negative bacteria [93].

Mock privet, as a non-domesticated variety, generally contains higher levels of these phenolic compounds compared to cultivated varieties, likely due to natural selection and greater genetic diversity. The chemical stability of these compounds enables prolonged antimicrobial effects, further supporting their potential use in antimicrobial formulations.

In contrast, it was given in the literature that rosemary, lemon balm and sea fennel exhibit antimicrobial properties primarily due to the presence of volatile components in their essential oils, such as 1,8-cineole, thymol, carvacrol and citral. Although these compounds can be effective against certain microorganisms, their volatility and lower chemical stability in extract-based preparations may limit their efficacy [94]. Indeed, HPLC analysis of the extracts displays that carvacrol is present in olive, mock privet and in sea fennel flower, while some were also found as VOCs in sea fennel flower, leaf and rosemary. However, the amount was not sufficient to exert an antimicrobial effect.

It is difficult to compare the obtained results to the literature data, since to our knowledge, there were no similar studies conducted so far combining tested extracts with given fish pathogens. Recently, Al-Rimawi et al. [95] showed that the use of 0.2% olive leaf extracts, rich in oleuropein, resulted in complete inhibition of microbial growth of *Pseudomonas aeruginosa*, *Staphylococcus aureus* and *Escherichia coli* during the fourth week of storage in a growth-promotion antimicrobial test. However, in the present study, neither olive leaf nor mock privet extracts showed activity against *P. Aeruginosa*. Inactivity might be because, for some bacterial strains, a reduction can be achieved only after a certain period of exposure [95].

Olive leaf extract was also shown to positively affect infections in fish caused by *A. hydrophila* [96]. This finding may be related to the activity shown in the present study as well. Bisignano et al. [97] showed MIC of 125 µg/mL for oleuropein against *V. alginolyticus*.

**Table 3:** Comparison of the antioxidant activity measured via DPPH and FRAP methods with available literature data.

Study (Reference)	Conditions	Antioxidant Activity–DPPH				Antioxidant Activity–FRAP			
		Sea Fennel Leaf		Sea Fennel Flower		Sea Fennel Leaf		Sea Fennel Flower	
		W	HA	W	HA	W	HA	W	HA
**Present work**	**MAE, P/S 1:20, 500 W, 10 min**	**44.81 ± 0.43%**	**48.44 ± 0.26%**	**84.38 ± 0.92%**	**78.77 ± 0.60%**	**105.77 ± 5.94 mg_AAE_/g**	**55.16 ± 0.00 mg_AAE_/g**	**219.67 ± 10.55 mg_AAE_/g**	**96.83 ± 6.54 mg_AAE_/g**
**[31]**	MAE, P/S 1:10, 500 W	*x*	*x*	*x*	*x*	*x*	185.01	*x*	*x*
**[48]**	Infusion, 1:20	IC_50_ 148–298 µg/mL	*x*	310	*x*	48–67	*x*	70 μM Fe^2+^	*x*
**[98]**	MAE, P/S 1:10, 30 min, 500 W	29–36%	*x*	*x*	*x*	12–14.36 mM Fe^2+^	*x*	*x*	*x*
**[99]**	Infusion, P/S 1:25	*x*	*x*	*x*	*x*	*x*	EC_50_ 0.152 mg/mL	*x*	*x*
**[100]**	Sequiential extraction, P/S 1:10	81	132 µg_Trolox_/mg	*x*	60	147	231 µg_Trolox Eq_/mg	122	232
**Rosemary**									
		**W**		**HA**		**W**		**HA**	
**Present work**		**67.62 ± 3.24%**		**16.83 ± 0.34%**		**57.25 ± 0.66 mg_AAE_/g**		**52.33 ± 0.00 mg_AAE_/g**	
**[60]**	Conv., 50:50 E:W, 24 h	*x*		20.6 ± 1.1 µg/mL		*x*		*x*	
	MAE, 50:50 E:W, 50 °C, 1 h	*x*		19.2 ± 0.7 µg/mL		*x*		*x*	
**[63]**	Stirring extraction	*x*		110.1 ± 6.61 µmol_AAE_/g		*x*		161.95 ± 3.56 µmol_AAE_/g	
	UAE	*x*		97.96 ± 3.33 µmol_AAE_/g		*x*		127.69 ± 6.51 µmol_AAE_/g	
**[101]**	Maceration	1.04 ± 0.06 mg_BHT_/mL		*x*		0.52 ± 0.05 mg FeSO_4_ × 7 H_2_O/mL		*x*	
**[102]**	Conv. Ex., 16 h	105 mg_GAE_/g		*x*		125 mgAsc/g		*x*	
**[59]**	Conv. Ex., 70 °C, 30 min	*x*		4745.72 ± 0.47 µmol_TE_/g		*x*		180.09 ± 0.01 µmol Fe^2+^/g	
**Olive**									
**Present work**		**67.89 ± 130%**		**9.78 ± 0.51%**		**38.62 ± 0.42 mg_AAE_/g**		**50.45 ± 0.69 mg_AAE_/g**	
**[91]**	Conv. Ex., 24 h	7.48 ± 0.39 μg/mL		10.88 ± 0.22 μg/mL		153.72 ± 2.70 mg_AAE_/g		258.76 ± 6.69 mg_AAE_/g	
**[103]**	Conv. Ex., 60 °C, 15 min	0.65 ± 0.09 mmol FeSO_4_ × 7 H_2_O/mL		*x*		0.28 ± 0.00 mmol_Trolox_/g		*x*	
	MAE, 400 W, 60 °C, 5 min	0.83 ± 0.08 mmol FeSO_4_ × 7 H_2_O/mL		*x*		0.39 ± 0.08 mmol_Trolox_/g		*x*	
**Mock privet**									
**Present work**		**21.78 ± 1.51%**		**13.31 ±0.15%**		**37.33 ± 2.65 mg_AAE_/g**		**74.95 ± 0.08 mg_AAE_/g**	
**[37]**	UAE 30 min, 30 °C	*x*		111.92 ± 1.38 mg_TE_/g		*x*		60.92 ± 0.24 mg_TE_/g	
	MAE, 30 min, 40 °C	*x*		102.56 ± 0.65 mg_TE_/g		*x*		53.60 ± 0.45 mg_TE_/g	
**[104]**	Electrothermal heating ex., 70 °C	*x*		62.48 ± 3.71 IC50 µg/mL		*x*		32.66 ± 4.27 mg_TE_/g	
**[105]**	Conv. Ex.	*x*		IC_50_ 29.0 µg/mL		*x*		*x*	
**Lemon balm**									
**Present work**		**50.89 ±3.85%**		**55.63 ± 0.37%**		**142.44 ± 5.42 mg_AAE_/g**		**224.34 ± 6.00 mg_AAE_/g**	
**[88]**	Extraction in an oven at 40 °C for 24 h	IC_50_ 62.83 ± 0.80 µg/mL		*x*		*x*		250.39 ± 38.80 µmol_Trolox_/L	
**[106]**	Extraction uder reflux of solvent, 2.5 h	IC_50_ 4.91 ± 0.49		IC_50_ 4.76 ± 0.48		329.06 ± 23.75 mg_AAE_/g		294.39 ± 11.67 mg_AAE_/g	
**[107]**	UAE, 540 W, 4 min, 50 °C	2006.9 ± 83.5 µmol_TE_/g		*x*		*x*		*x*	
**[108]**	UAE, 2 min, 30 °C	470 mg_GAE_/g		*x*		*x*		680 mg_GAE_/g	
**[109]**	Water bath extraction for 10 h, 45 °C	0.58 ± 0.01 mmol_TE_/g extract		*x*		*x*		0.26 ± 0.01 mmol TE/g extract	

P/S—plant/solvent ratio; Conv. Ex.—conventional extraction; UAE—ultrasound-assisted extraction; MAE—microwave-assisted extraction; *x*—not known.

**Table 4:** Antimicrobial susceptibility of marine pathogens.

Microorganisms	Sea Fennel Leaf	Sea Fennel Flower	Rosemary	Olive	Mock Privet	Lemon Balm
	Zone of Inhibition in mm (Mean ± SE)					
*V. fisheri*	24.0 ± 0.3	/	18.0 ± 0.3	25.0 ± 0.1	21.0 ± 0.1	30.0 ± 0.2
*V. alginolyticus*	/	/	/	13.0 ± 0.2	17.0 ± 0.1	/
*M. lacunata*	/	14.5 ± 0.3	/	28.0 ± 0.1	30.0 ± 0.2	/
*Y. ruckeri*	/	/	/	/	/	/
*P. aeruginosa*	/	/	/	/	/	/
*A. hydrophila*	/	20.0 ± 0.1	/	26.0 ± 0.1	15.0 ± 0.2	/

For all analysis, 30 mg of lyophilized water extracts was used.

## 4. Conclusions

In the present study, microwave-assisted extraction of bioactive compounds from plants was carried out with water or a mixture of ethanol and water as solvents. Higher concentrations of total phenolic and total flavonoid content were obtained in the hydroalcoholic extracts. Furthermore, the extracts were analyzed via HPLC and HS-SPME/GC–MS in order to obtain a detail overview of the phenols and volatile organic compounds presented in the plant extracts. The most abundant phenolic compounds detected in plant extracts were rosmarinic acid and chlorogenic acid, and among the flavonoids, naringin and oleuropein. Considering the analyzed volatile organic compounds, terpenoids, alcohols, aldehydes and ketones were found in the extracts. The highest value of antioxidant activity determined via the FRAP method was obtained for the sea fennel flower water extract and the lemon balm hydroalcoholic extract. Also, the results for DPPH radical inhibition were similar across all plant extracts and did not correlate with TPC. The extracts prepared in this research did not show any significant microbial activity, and the reason can be insufficient concentration of different phenolic compounds. However, it is not impossible that if used in real food products, especially in synergy with naturally occurring carriers and encapsulants like biopolymer films, they may influence the product shelf life. Therefore, currently obtained knowledge on the antioxidant properties, profile of bioactive and antimicrobial activity will serve as a bases for applying these extracts in biobased edible coatings for fish preservation. In addition, this research provides a comprehensive characterization of volatile compounds in plant extracts, a topic that is often overlooked in the existing literature. It compares the antioxidant and antimicrobial activities of these extracts against specific fish pathogens, filling a critical gap in scientific understanding, particularly with regard to applications in the food and packaging industries. In addition, this study provides more detailed insights than conventional methods using an advanced analytical technique. An important limitation is the insufficient antimicrobial activity of the extracts, which suggests future optimisation to improve their efficacy.

## Figures and Tables

**Table 1:** Total phenol content (TPC) and total flavonoid content (TFC) of water (W) and hydroalcoholic (HA) plant extracts.

Plant Material	Extract	TPC		TFC	
		mg_GAE_/L	mg_GAE_/g_plant_	mg_QE_/L	mg_QE_/g_plant_
**Sea fennel leaf**	W	648.48 ± 50.07 ^f^	12.97 ± 1.00 ^g^	122.30 ± 4.16 ^g^	2.45 ± 0.08 ^g^
	HA	854.24 ± 2.54 ^e,f^	17.085 ± 0.25 ^e,f,g^	333.50 ± 4.69 ^f^	6.67 ± 0.09 ^f^
**Sea fennel flower**	W	1585.58 ± 62.13 ^b^	31.711 ± 1.24 ^c^	414.09 ± 32.21 ^e,f^	8.28 ± 0.64 ^e,f^
	HA	973.64 ± 27.74 ^c,d,e^	19.47 ± 0.56 ^d,e,f^	1794.63 ± 22.87 ^a^	35.89 ± 0.46 ^a^
**Rosemary**	W	836.00 ± 13.83 ^e,f^	16.72 ± 0.28 ^f,g^	123.50 ± 6.95 ^g^	2.47 ± 0.14 ^g^
	HA	3321.21 ± 189.02 ^a^	66.42 ± 3.78 ^a^	603.53 ± 19.02 ^c^	12.07 ± 0.38 ^c^
**Olive**	W	204.55 ± 7.10 ^g^	4.09 ± 0.14 ^h^	93.70 ± 7.07 ^g^	1.87 ± 0.14 ^g^
	HA	904.55 ± 52.20 ^d,e^	18.09 ± 1.04 ^e,f,g^	470.13 ± 43.93 ^d,e^	9.40 ± 0.88 ^d,e^
**Mock privet**	W	1044.15 ± 34.13 ^c,d,e^	49.51 ± 0.68 ^b^	161.03 ± 16.18 ^g^	3.22 ± 0.32 ^g^
	HA	906.97 ± 9.33 ^d,e^	18.14 ± 0.19 ^e,f,g^	771.27 ± 69.62 ^b^	15.43 ± 1.39 ^b^
**Lemon balm**	W	1126.94 ± 28.43 ^c,d^	22.54 ± 3.78 ^d,e^	521.99 ± 9.21 ^c,d^	10.44 ± 0.18 ^c,d^
	HA	1199.09 ± 153.52 ^c^	23.98 ± 3.07 ^d^	582.16 ± 26.16 ^c^	11.64 ± 0.52 ^c^

Different exponents (^a–g^) within the same column indicate significant differences among samples (*p* < 0.05).

**Table 2:** Profile of phenolic compounds detected via HPLC analysis of water and hydroalcoholic extracts of sea fennel leaves and flowers, rosemary, olive, mock privet and lemon balm leaves.

Compound		Water Extracts						Hydroalcoholic Extracts					
		Sea Fennel Leaf	Sea Fennel Flower	Rosemary	Olive	Mock Privet	Lemon Balm	Sea Fennel Leaf	Sea Fennel Flower	Rosemary	Olive	Mock Privet	Lemon Balm
**Caffeic acid**	mg/L extract	1.977 ± 0.020 ^j,E^	6.120 ± 0.194 ^g,h,i,B^	0.163 ± 0.001 ^l,H^	3.290 ± 0.017 ^c,D^	1.532 ± 0.054 ^f,g,F^	0.139 ± 0.001 ^j,H^	5.205 ± 0.338 ^i,j,C^	6.615 ± 0.010 ^j,A^	n.d. ^l,H^	5.931 ± 0.015 ^d,e,f,g,h,B^	2.034 ± 0.010 ^k,l,E^	0.604 ± 0.008 ^k,G^
**Coumaric acid**		10.054 ± 0.082 ^e,D^	65.146 ± 0.120 ^d,B^	0.416 ± 0.012 ^j,k,G,H^	0.479 ± 0.008 ^f,g,h,G^	0.208 ± 0.015 ^g,G,H,I^	0.131 ± 0.003 ^j,H,I^	12.219 ± 0.053 ^g,C^	93.357 ± 0.362 ^c,A^	n.d. ^l,I^	2.149 ± 0.033 ^f,g,h,F^	2.538 ± 0.004 ^E^	n.d. ^k,I^
**Ellagic acid**		0.120 ± 0.000 ^n,o,G^	0.587 ± 0.029 ^i,C^	0.047 ± 0.001 ^m,H^	0.412 ± 0.000 ^g,h,E^	0.057 ± 0.002 ^g,H^	0.041 ± 0.011 ^k,H^	0.353 ± 0.002 ^k,F^	0.492 ± 0.000 ^l,D^	0.409 ± 0.002 ^k,l,E^	1.250 ± 0.003 ^g,h,A^	0.451 ± 0.003 ^l,m,D,E^	0.653 ± 0.043 ^k,B^
**Ferulic acid**		10.695 ± 0.017 ^d,G^	78.958 ± 0.444 ^c,A^	0.826 ± 0.008 ^h,L^	2.157 ± 0.002 ^d,K^	14.680 ± 0.070 ^b,F^	5.097 ± 0.016 ^b,J^	31.376 ± 0.085 ^e,E^	66.872 ± 0.049 ^e,B^	9.463 ± 0.005 ^e,f,H^	7.897 ± 0.032 ^d,e,f,g,h,I^	55.149 ± 0.112 b,^C^	42.956 ± 0.296 ^b,D^
**Protocatechuic acid**		1.919 ± 0.009 ^j,k,G^	3.394 ± 0.057 ^g,h,i,F^	1.068 ± 0.053 ^g,H^	0.704 ± 0.001 ^e,f,g,h,H^	1.044 ± 0.009 ^j,^	1.100 ± 0.163 ^f,H^	8.698 ± 0.525 ^h,E^	15.302 ± 0.003 ^hA^	9.765 ± 0.009 ^e,D^	8.269 ± 0.439 ^d,e,f,g,E^	11.633 ± 0.008 g,^C^	12.608 ± 0.041 ^e,f,B^
**4-hydroxybenzoic acid**		4.689 ± 0.000 ^h,E^	9.774 ± 0.239 ^g,B^	4.258 ± 0.005 ^d,E^	0.253 ± 0.001 ^g,h,H^	1.171 ± 0.027 ^g,G^	0.385 ± 0.019 ^i,H^	8.971 ± 0.591 ^h,C^	4.724 ± 0.238 ^k,E^	8.140 ± 0.000 ^g,D^	0.710 ± 0.005 ^g,h,G,H^	18.160 ± 0.005 ^f,A^	2.266 ± 0.023 ^j,F^
**Caffeoylmalic acid**		2.669 ± 0.077 ^i,D^	6.083 ± 0.016 ^g,h,i,A^	0.335 ± 0.005 ^k,G^	1.475 ± 0.005 ^d,e,f,E^	3.001 ± 0.021 ^f,C^	0.869 ± 0.002 ^g,F^	0.763 ± 0.116 k,F	0.339 ± 0.007 ^l,G^	3.856 ± 0.044 ^j,B^	4.004 ± 0.189 ^e,f,g,h,B^	2.572 ± 0.002 ^j,k,D^	2.504 ± 0.053 ^j,D^
**Gallic acid**		1.264 ± 0.003 ^l,m,G^	2.738 ± 0.006 ^h,i,E^	0.520 ± 0.001 ^i,j,I^	0.925 ± 0.002 ^e,f,g,h,H^	1.437 ± 0.023 ^g,F^	0.506 ± 0.004 ^h,I^	3.769 ± 0.009 ^j,C^	3.574 ± 0.040 ^k,D^	2.805 ± 0.009 ^j,E^	3.987 ± 0.017 ^e,f,g,h,B^	9.254 ± 0.106 ^h,A^	3.484 ± 0.127 ^i,D^
**Chlorogenic acid**		**106.464 ± 0.475 ^a,D^**	683.137 ± 0.105 ^b,B^	0.795 ± 0.002 ^h,I^	0.836 ± 0.001 ^e,f,g,h,I^	0.931 ± 0.020 g^,I^	1.047 ± 0.003 ^f,I^	329.939 ± 0.023 ^b,C^	765.234 ± 0.231 ^b,A^	6.468 ± 0.005 ^h,F^	5.557 ± 0.001 ^d,e,f,g,h,G^	4.608 ± 0.001 ^i,H^	12.728 ± 0.048 ^e,E^
**Rosmarinic acid**		7.760 ± 0.228 ^f,J^	18.022 ± 0.044 ^f,I^	61.369 ± 0.142 ^b,E^	3.887 ± 0.022 ^b,c,K^	4.815 ± 0.250 ^e,K^	**107.307 ± 0.154 ^a,C^**	49.474 ± 1.128 ^d,F^	74.233 ± 0.076 ^d,D^	326.553 ± 1.299 ^b,B^	21.772 ± 0.195 ^b,H^	36.196 ± 0.514 ^d,G^	**1282.644 ± 1.063 ^a,A^**
**Vanilic acid**		2.787 ± 0.035 ^i,F^	5.745 ± 0.056 ^g,h,i,C^	3.321 ± 0.003 ^e,E^	0.746 ± 0.006 ^e,f,g,h,H^	0.185 ± 0.001 ^g,I^	2.497 ± 0.003 ^e,F^	7.494 ± 0.405 ^h,i,B^	1.191 ± 0.017 ^l,G^	12.336 ± 0.021 ^d,A^	3.556 ± 0.035 ^f,g,h,E^	5.095 ± 0.083 ^i,D^	2.800 ± 0.010 ^i,j,F^
**Naringin**		90.838 ± 0.013 ^b,E^	**4039.836 ± 9.638 ^a,A^**	**75.755 ± 0.007 ^a,F^**	4.784 ± 0.022 ^b,J^	8.566 ± 0.033 ^c,J^	4.829 ± 0.047 ^c,J^	**366.263 ± 0.735 ^a,C^**	**3228.736 ± 2.321 ^a,B^**	**337.536 ± 0.340 ^a,D^**	21.080 ± 0.214 ^b,c,I^	52.622 ± 5.208 ^c,G^	36.116 ± 0.034 ^c,H^
**Kaempferol**		1.515 ± 0.000 ^k,l,E^	3.475 ± 0.000 ^g,h,i,D^	0.384 ± 0.018 ^k,H^	0.540 ± 0.000 ^f,g,h,F^	0.442 ± 0.000 ^g,G^	0.520 ± 0.001 ^h,F^	11.856 ± 0.000 ^g,C^	11.861 ± 0.000 ^i,C^	12.222 ± 0.055 ^d,A^	11.932 ± 0.004 ^d,e,B^	11.859 ± 0.000 ^g,C^	11.871 ± 0.000 ^f,C^
**Quercetin**		0.431 ± 0.000 ^n,J^	0.986 ± 0.001 ^i,H^	1.223 ± 0.002 ^f,G^	0.489 ± 0.002 ^f,g,h,I^	0.238 ± 0.000 ^g,L^	0.400 ± 0.001 ^i,K^	3.390 ± 0.000 ^j,F^	3.506 ± 0.001 ^k,D^	8.908 ± 0.002 ^e,f,g,A^	3.400 ± 0.000 ^f,g,h,E^	3.773 ± 0.002 ^i,j,k,C^	7.303 ± 0.011 ^h,B^
**Myricetin**		0.057 ± 0.001 ^n,o,F^	8.938 ± 0.002 ^g,h,B^	0.962 ± 0.006 ^g,E^	2.170 ± 0.001 ^d,D^	0.728 ± 0.001 ^g,E^	n.d. ^k,F^	13.514 ± 0.062 ^g,F^	0.972 ± 0.019 ^l,E^	8.691 ± 0.075 ^f,g,B^	8.643 ± 0.013 ^d,e,f,g,B^	4.413 ± 0.465 ^i,j,C^	n.d. ^k,F^
**Quercetin-3-glucoside**		6.897 ± 0.042 ^g,H^	45.744 ± 0.256 ^e,B^	0.557 ± 0.004 ^i,F^	1.618 ± 0.197 ^d,e,J^	6.788 ± 0.009 ^d,H^	2.899 ± 0.009 ^d,J^	26.580 ± 0.218 ^f,E^	48.338 ± 0.012 ^f,A^	12.570 ± 0.058 ^d,F^	10.292 ± 0.034 ^d,e,f,G^	36.809 ± 0.211 ^d,C^	31.230 ± 0.243 ^d,D^
**Rutin trihydrate**		1.058 ± 0.030 ^m,E^	4.050 ± 0.047 ^g,h,i,D^	0.221 ± 0.001 ^l,F^	0.757 ± 0.001 ^e,f,g,h,E,F^	0.339 ± 0.004 ^g,E,F^	0.390 ± 0.013 ^i,E,F^	7.992 ± 0.849 ^h,C^	7.355 ± 0.002 ^j,C^	9.486 ± 0.021 ^e,f,B^	9.711 ± 0.054 ^d,e,f,A,B^	9.374 ± 0.018 ^h,B^	10.313 ± 0.093 ^g,A^
**Oleuropein**		14.682 ± 0.264 ^c,H^	66.914 ± 1.575 ^d,F^	4.697 ± 0.049 ^c,H,I^	**556.738 ± 1.503 ^a,C^**	**325.065 ± 2.251 ^a,D^**	0.795 ± 0.028 ^g,I^	144.881 ± 3.039 ^c,E^	37.420 ± 0.803 ^g,G^	15.091 ± 0.117 ^c,H^	**2004.922 ± 12.079 ^a,B^**	**2318.626 ± 0.264 ^a,A^**	7.201 ± 0.140 ^h,H,I^
**Luteolin**		n.d. ^o,E^	0.042 ± 0.001 ^i,E^	0.562 ± 0.001 ^i,D^	1.223 ± 0.001 ^d,e,f,g,B^	0.700 ± 0.010 ^g,C^	0.023 ± 0.000 ^k,E^	n.d. ^k,E^	n.d. ^l,E^	n.d. ^l,E^	n.d. ^h,E^	2.324 ± 0.159 ^k,l,A^	0.669 ± 0.003 ^k,C,D^
**Apigenin**		n.d. ^o,F^	1.388 ± 0.017 ^i,B^	0.211 ± 0.004 ^l,D^	0.068 ± 0.000 ^h,E^	0.055 ± 0.000 ^g,F^	n.d. ^k,F^	n.d.^.k,F^	n.d. ^l,F^	1.264 ± 0.003 ^k,C^	n.d. ^h,F^	n.d. ^m,F^	2.055 ± 0.018 ^j,A^
**Carvacrol**		n.d. ^o,D^	n.d. ^i,D^	n.d. ^m,D^	n.d. ^h,D^	n.d. ^g,D^	n.d. ^k,D^	n.d. ^k,D^	10.771 ± 0.373 ^i,C^	n.d. ^l,D^	13.423 ± 0.244 ^c,d,B^	30.017 ± 1.282 ^e,A^	n.d. ^k,D^

Bolded—values corresponding to the compound measured in the highest concentration in the given plant. Different exponents within the same column (^a–o^) and row (^A–L^) indicate significant differences among samples (*p* < 0.05).

## Data Availability

All of the data is contained within the article and Supplementary File.

## Supplementary Materials

The following supporting information can be downloaded at https://www.mdpi.com/article/10.3390/antiox14080906/s1, Table S1: Volatile organic compounds presented in sea fennel leaf extracts detected via HS-SPME/GC–MS; Table S2. Volatile organic compounds presented in sea fennel flower extracts detected via HS-SPME/GC–MS; Table S3. Volatile organic compounds presented in rosemary extracts detected via HS-SPME/GC–MS; Table S4. Volatile organic compounds presented in olive leaf extracts detected via HS-SPME/GC–MS; Table S5. Volatile organic compounds presented in mock privet leaf extracts detected via HS-SPME/GC–MS; Table S6. Volatile organic compounds presented in lemon balm leaf extracts detected via HS-SPME/GC–MS. Figure S1. Photos of the used plants: (a) Sea fennel, (b) Rosemary, (c) Olive, (d) Mock privet, and (e) Lemon balm; Figure S2. The scheme of all the steps and analysis which were performed in this work; Figure S3. The predominant polyphenol compounds detected by HPLC in water and hydroalcoholic plant extracts: (a) Sea fennel leaf; (b) Sea fennel flower; (c) Rosemary leaf; (d) Olive leaf; (e) Mock privet leaf and (f) Lemon balm leaf; Figure S4. Selected polyphenol compounds detected by HS-SPME-GC/MS in water and hydroalcoholic plant extracts: (a) Sea fennel leaf; (b) Sea fennel flower; (c) Rosemary leaf; (d) Olive leaf; (e) Mock privet leaf and (f) Lemon balm leaf.
