# Supplementary material for: Properties of Plant Extracts from Adriatic Maritime Zone for Innovative Food and Packaging Applications: Insights into Bioactive Profiles, Protective Effects, Antioxidant Potentials and Antimicrobial Activity
Source: Antioxidants (Basel). 2025 Jul 24;14(8):906. doi: 10.3390/antiox14080906 (PMC12383195; doi:10.3390/antiox14080906)
Supplement: Supplementary file 1 [file antioxidants-14-00906-s001.zip › antioxidants-3723752-supplementary.pdf]

SUPPLEMENTARY

Properties of plant extracts from Adriatic maritime zone for innovative food and packaging applications: Insights into Bioactive profiles, Protective Effects, Antioxidant Potentials and Antimicrobial Activity

Petra Babić, Tea Sokač Cvetnić, Iva Čanak, Mia Dujmović, Mojca Čakić Semenčić, Filip Šupljika, Zoja Vranješ, Frédéric Debeaufort, Nasreddine Benbettaieb, Emilie Descours and Mia Kurek

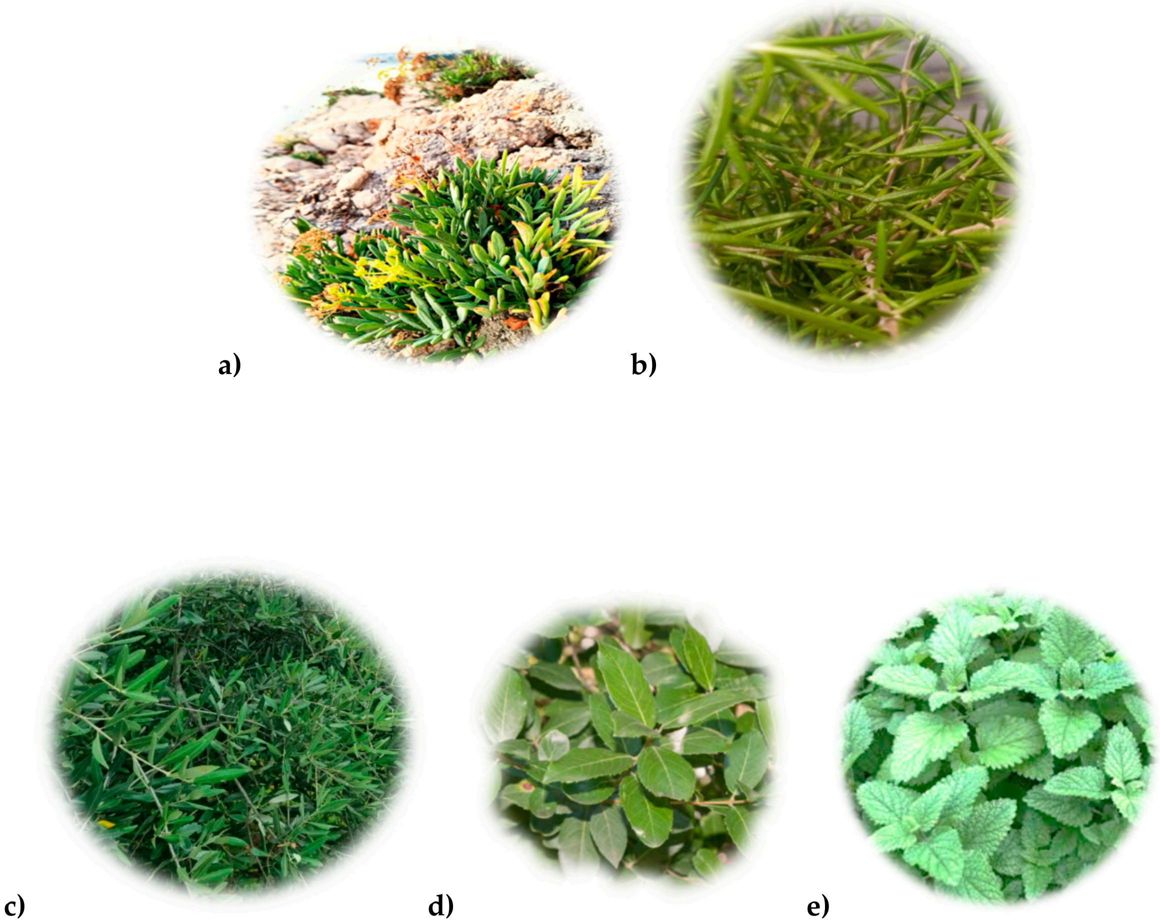

Supplementary Figure S1. Photos of the used plants: a) Sea fennel, b) Rosemary, c) Olive, d) Mock privet, and e) Lemon balm

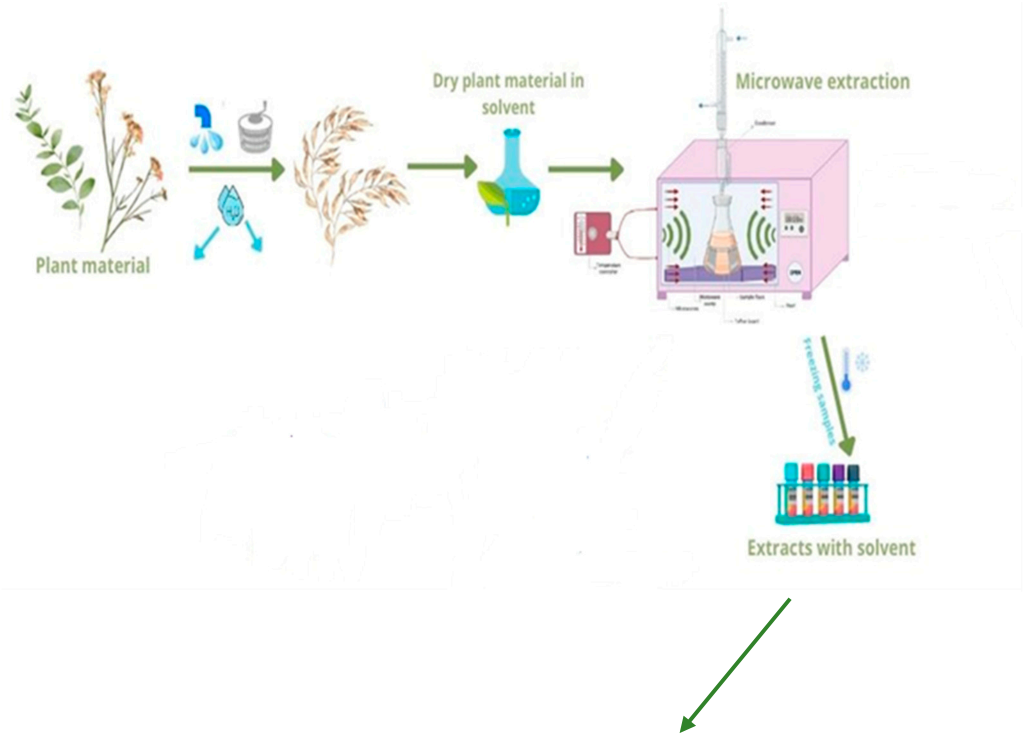

The analysis of extracts include:

1. Spectrophotometric analysis of total polyphenol content and total flavonoid content
2. HPLC analysis of phenolic compounds
3. GC-MS analysis for the determination of volatile compounds
4. Spectrophotometric analysis of total chlorophylls and total carotenoids
5. Determination of antioxidant activity using DPPH and FRAP method
6. Antimicrobial activity

Supplementary Figure S2. The scheme of all the steps and analysis which were performed in this work

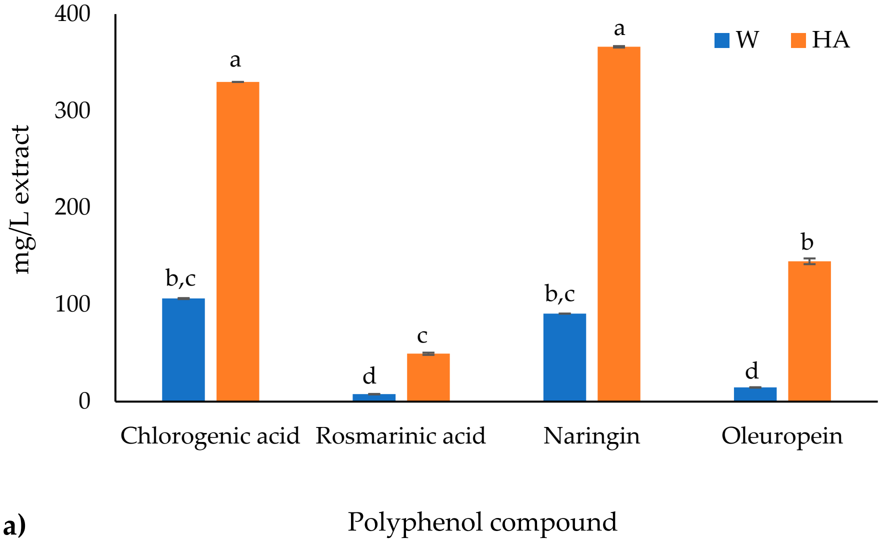

400
300
200
100
0
mg/L extract
W
HA
a
b,c
d
c
b,c
d
b
Chlorogenic acid
Rosmarinic acid
Naringin
Oleuropein
Polyphenol compound

a)

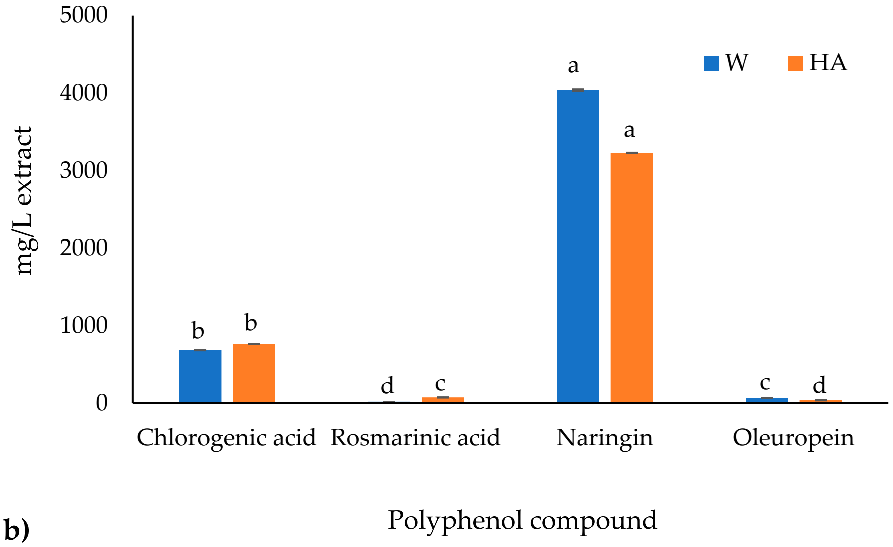

5000
4000
3000
2000
1000
0
mg/L extract
W
HA
b
d
c
a
c
d
Chlorogenic acid
Rosmarinic acid
Naringin
Oleuropein
Polyphenol compound

b)

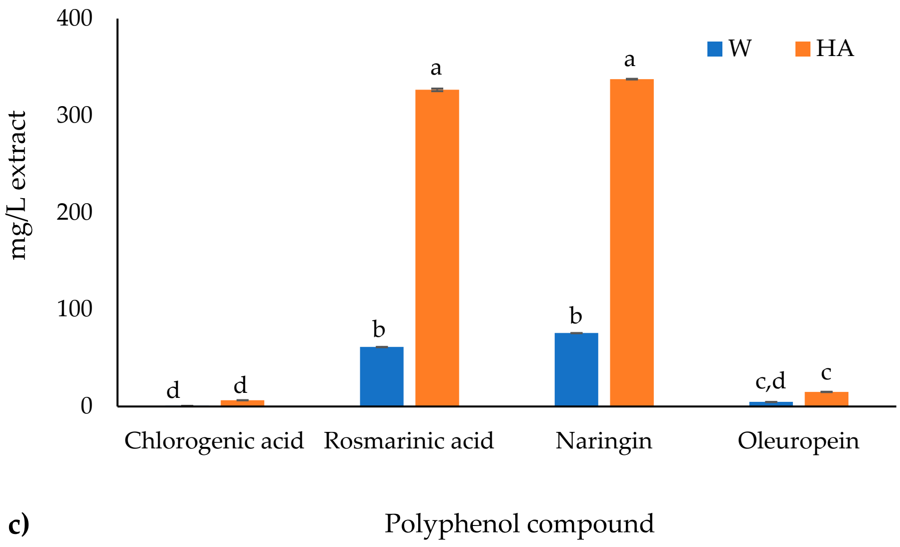

400
300
200
100
0
mg/L extract
W
HA
a
a
b
b
d
d
c,d
c
Chlorogenic acid
Rosmarinic acid
Naringin
Oleuropein
Polyphenol compound

c)

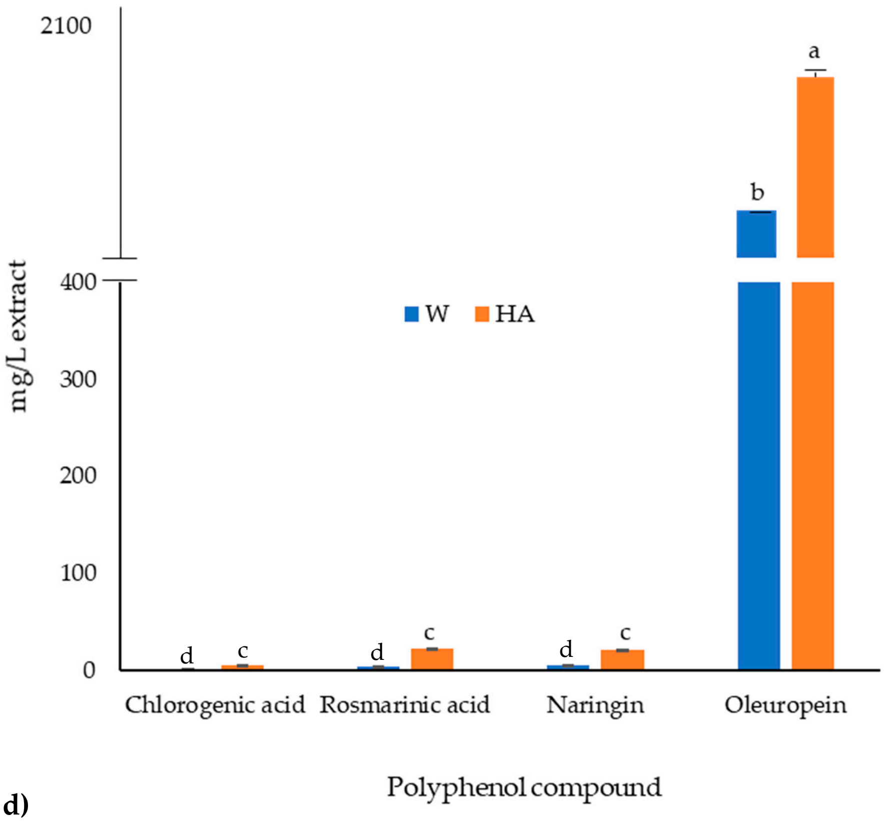

2100
400
300
200
100
0
mg/L extract
W
HA
a
b
d
c
d
c
d
c
Chlorogenic acid
Rosmarinic acid
Naringin
Oleuropein
Polyphenol compound

d)

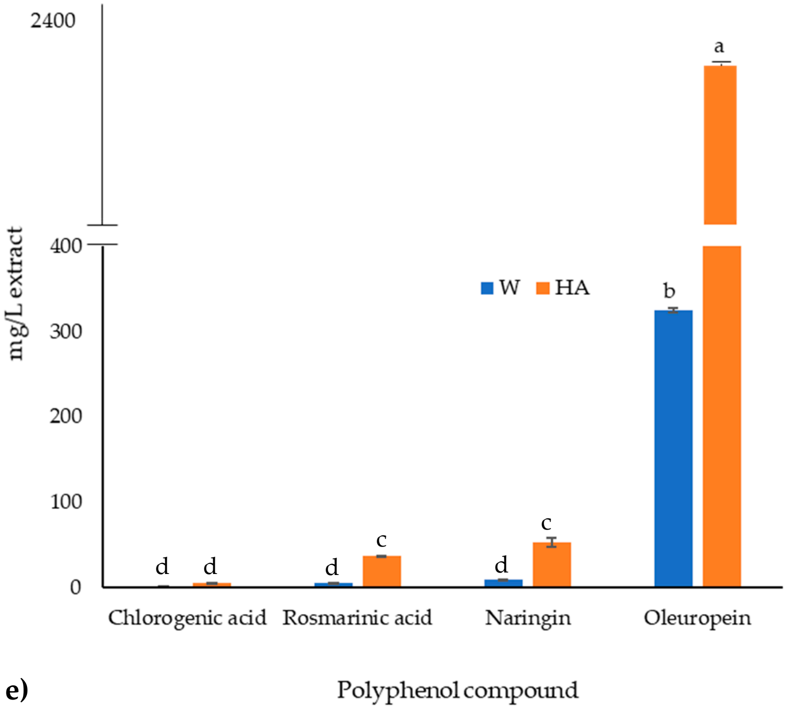

e)

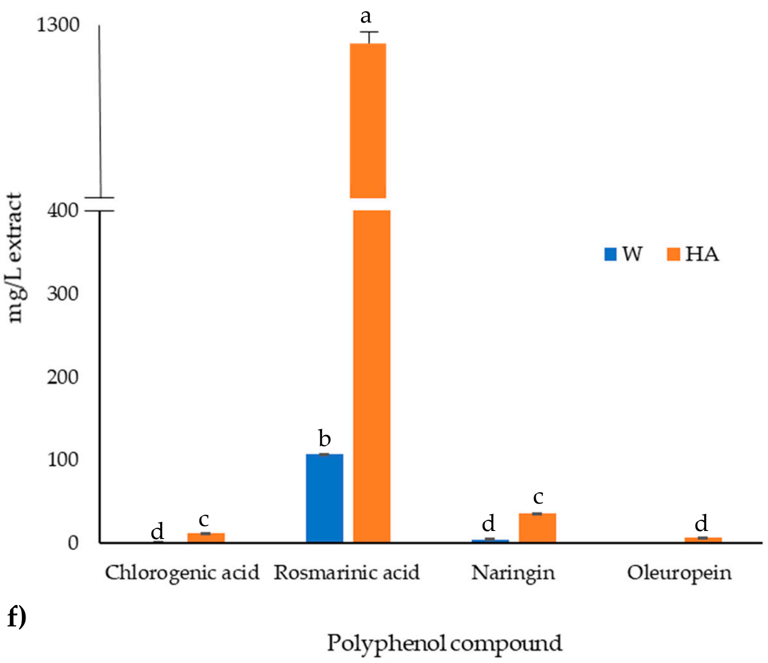

f)

Supplementary Figure S3. The predominant polyphenol compounds detected by HPLC in water and hydroalcoholic plant extracts: a) Sea fennel leaf; b) Sea fennel flower; c) Rosemary leaf; d) Olive leaf; e) Mock privet leaf and f) Lemon balm leaf

a)

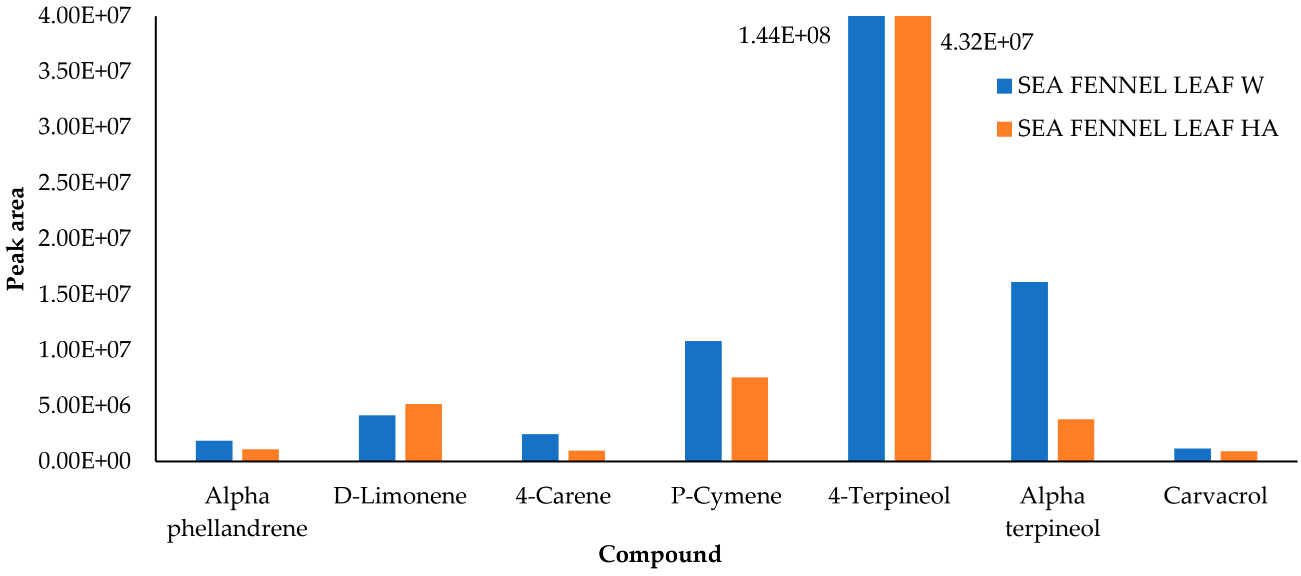
4.00E+07
3.50E+07
3.00E+07
2.50E+07
2.00E+07
1.50E+07
1.00E+07
5.00E+06
0.00E+00
Peak area
1.44E+08
4.32E+07
SEA FENNEL LEAF W
SEA FENNEL LEAF HA
Alpha phellandrene
D-Limonene
4-Carene
P-Cymene
4-Terpineol
Alpha terpineol
Carvacrol
Compound

b)

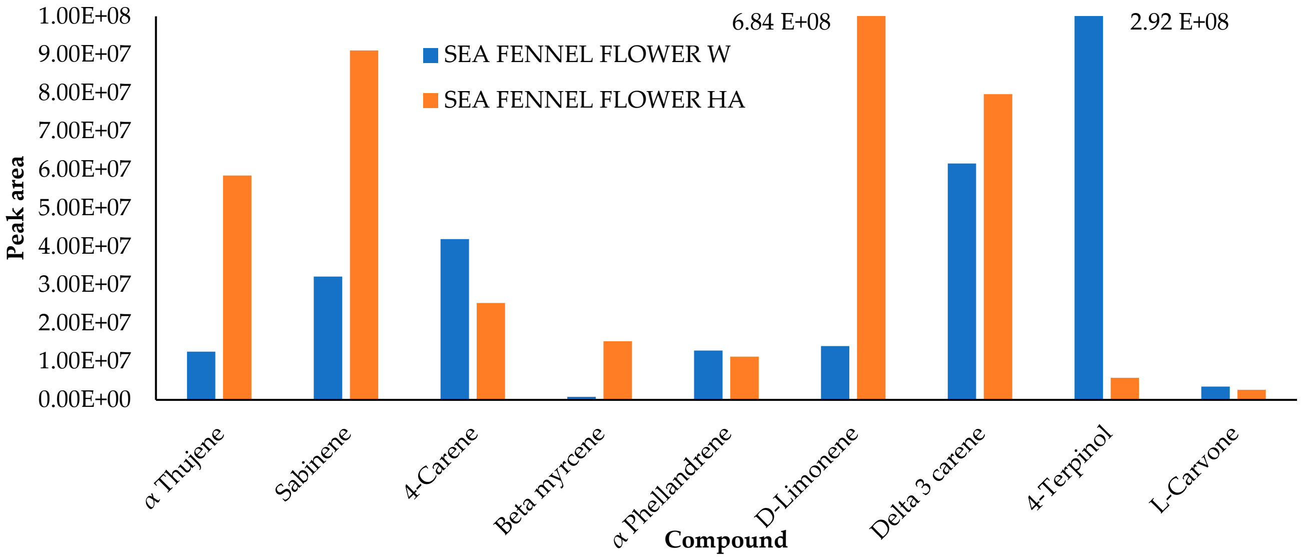
1.00E+08
9.00E+07
8.00E+07
7.00E+07
6.00E+07
5.00E+07
4.00E+07
3.00E+07
2.00E+07
1.00E+07
0.00E+00
Peak area
6.84 E+08
2.92 E+08
SEA FENNEL FLOWER W
SEA FENNEL FLOWER HA
α Thujene
Sabinene
4-Carene
Beta myrcene
α Phellandrene
D-Limonene
Delta 3 carene
4-Terpinol
L-Carvone
Compound

c)

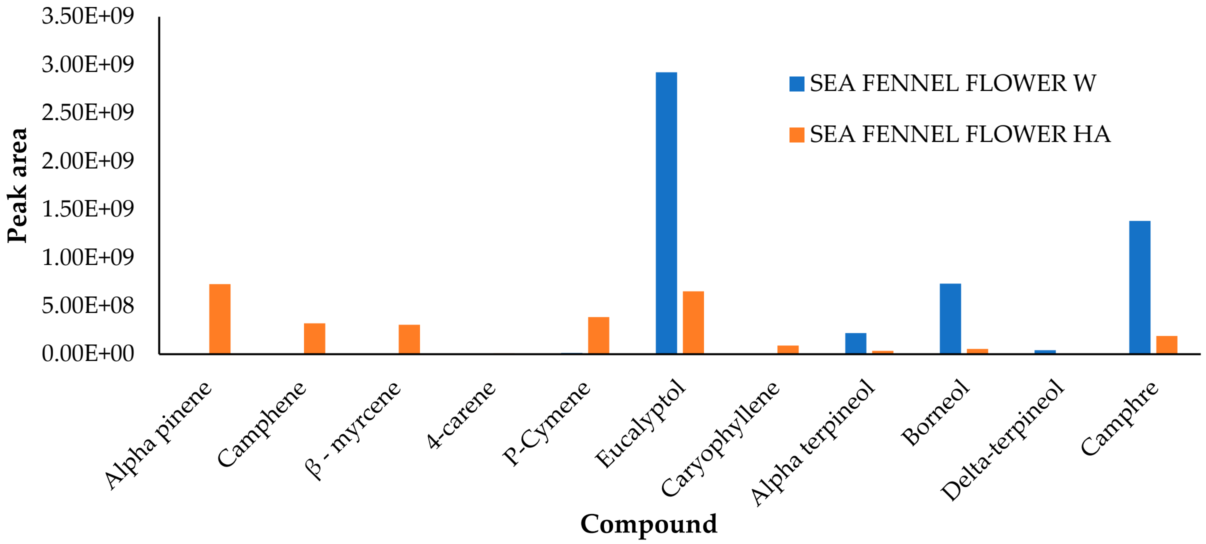

3.50E+09
3.00E+09
2.50E+09
2.00E+09
1.50E+09
1.00E+09
5.00E+08
0.00E+00
Peak area
SEA FENNEL FLOWER W
SEA FENNEL FLOWER HA
Alpha pinene
Camphene
β - myrcene
4-carene
P-Cymene
Eucalyptol
Caryophyllene
Alpha terpineol
Borneol
Delta-terpineol
Camphre
Compound

d)

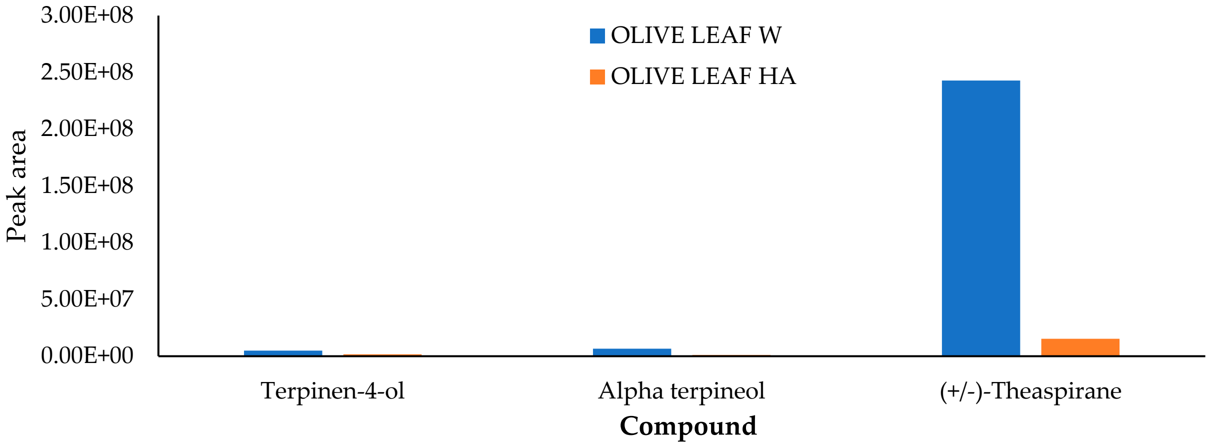

3.00E+08
2.50E+08
2.00E+08
1.50E+08
1.00E+08
5.00E+07
0.00E+00
Peak area
OLIVE LEAF W
OLIVE LEAF HA
Terpinen-4-ol
Alpha terpineol
(+/-)-Theaspirane
Compound

e)

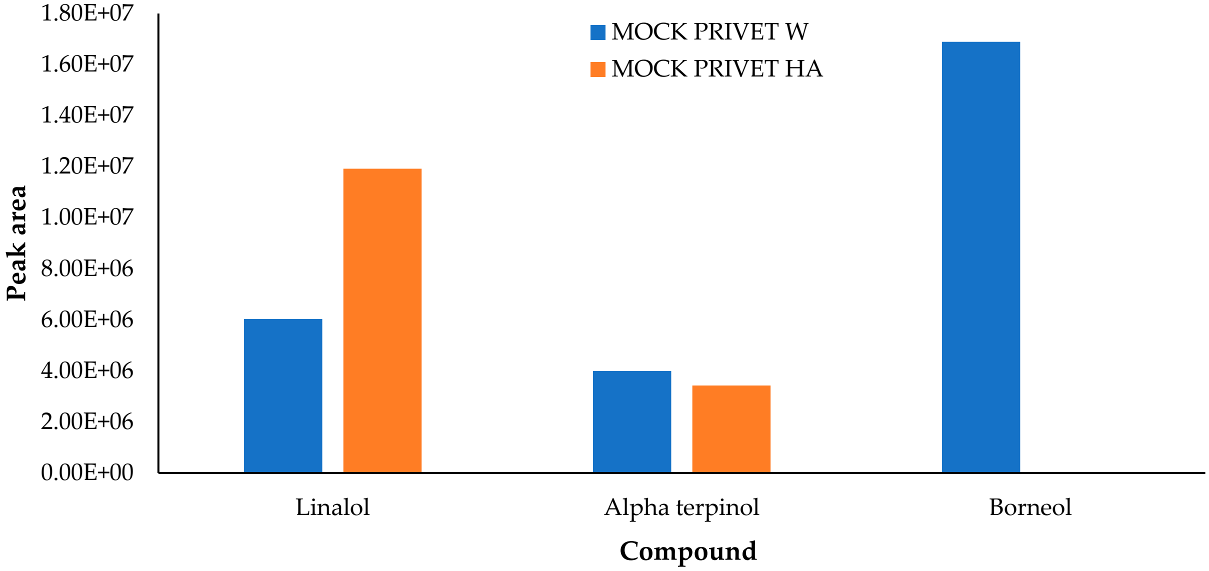

f)

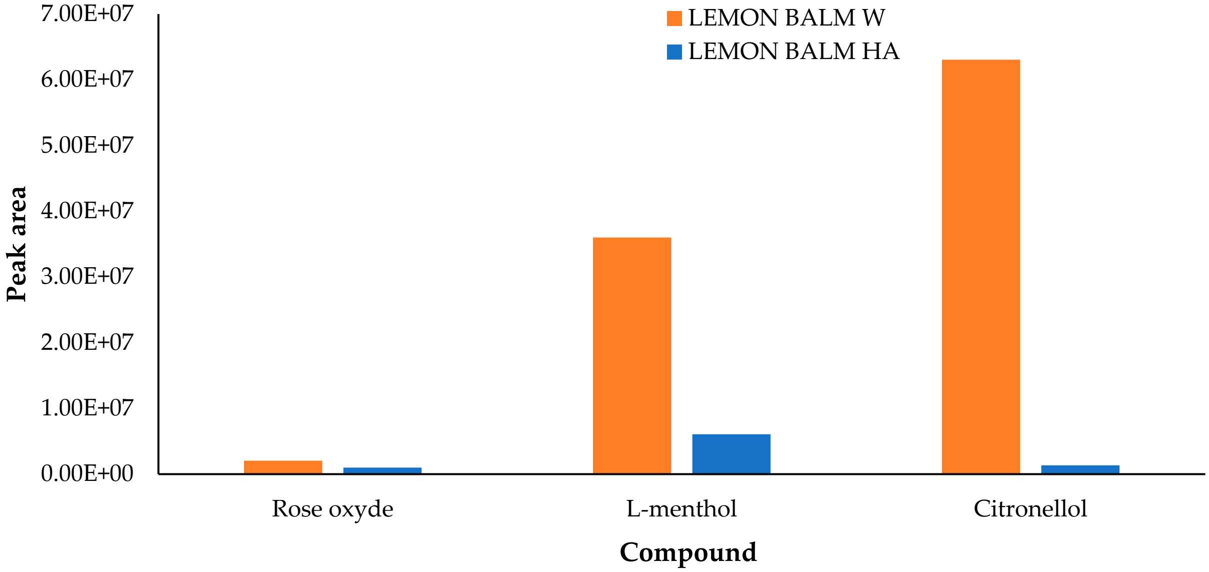

Supplementary Figure S4. Selected polyphenol compounds detected by HS-SPME-GC/MS in water and hydroalcoholic plant extracts: a) Sea fennel leaf; b) Sea fennel flower; c) Rosemary leaf; d) Olive leaf; e) Mock privet leaf and f) Lemon balm leaf

Supplementary Table S1. Volatile organic compounds presented in sea fennel leaf extracts detected by HS-SPME-GC/MS

WATER EXTRACT			HYDROALCOHOLIC EXTRACT		
**Name**	**CAS**	**% Area**	**Name**	**CAS**	**% Area**
**ALCOHOLS**					
Benzyl alcohol	100-51-6	4.63	Benzyl alcohol	100-51-6	1.10
Phenylethyl alcohol	60-12-8	0.11	Phenylethyl alcohol	60-12-8	1.67
2-methyl-1-Propanol	78-83-1	1.85	1-Octen-3-ol	3391-86-4	1.79
3,6-dimethyl-3-Heptanol	1573-28-0	0.13			
3-Octanol	589-98-0	0.11	**ALDEHYDES**		
2-ethyl-1-Hexanol	104-76-7	0.88	Hexanal	66-25-1	1.81
p-Cymen-7-ol	536-60-7	0.13	Heptanal	111-71-7	2.69
p-Menth-1-en-9-ol	13835-75-1	0.35	(E)-2-Heptenal	18829-55-5	1.40
3-methyl-1-Butanol	123-51-3	13.16	Octanal	124-13-0	11.88
Cis-p-mentha-1(7),8-dien-2-ol	374-16-8	4.22	Nonanal	124-19-6	1.24
Trans-p-mentha-1(7),8-dien-2-ol	374-16-7	0.72	Benzaldehyde	100-52-7	3.91
**KETONES**					
6-methyl-5-hepten-2-one	110-93-0	0.16	2-methyl-5-(1-methylethenyl)-cyclohexanone	7764-50-3	0.79
Acetoine	513-86-0	1.94	Sabina ketone	513-20-2	0.70
**TERPENOIDS**					
α - phellandrene	99-83-2	0.16	α -phellandrene	99-83-2	0.81
α - pinene	80-56-8	0.09	(-)-Spathulenol	77171-55-2	1.16
β- pinene	127-91-3	0.05	4-Terpeniol	562-74-3	31.75
4-Carene	2950-33-7	0.21	L-Menthol	2216-51-5	0.77
D-Limonene	5989-27-5	0.36	α terpeniol	10482-56-1	2.78
α - terpinene	99-86-5	0.34	Anethol	104-46-1	1.27
Sabinene	3387-41-5	0.35	4-Carene	2950-33-7	0.71
β -Phellandrene	555-10-2	0.43	D-Limonene	5989-27-5	3.81
P-Cymene	99-87-6	0.93	Sabinene	3387-41-5	0.79
Fenchone	1195-79-5	3.78	1,3,8-p-Menthatriene	18368-95-1	1.23
Fenchane	6248-88-0	1.18	p-entha-1,5,8-triene	21195-59-5	2.34
Linalool	78-70-6	4.41	P-Cymene	99-87-6	5.55
α tjuon	1125-12-8	0.10	2,4-thujadiene	36262-09-6	0.88
4-Terpineol	562-74-3	12.37	Spathulenol	6750-60-3	1.37
Trans-dihydrocarvone	5948-04-9	1.54	Carvacrol	499-75-2	0.67
Cis-dihydrocarvone	3792-53-8	0.14	(-)-Carvone	6485-40-1	0.54
Citral	5392-40-5	2.59	D - carvone	2244-16-8	0.97
α -terpineol	10482-56-1	1.38			
Thujone	546-80-5	0.39			

(+)-Borneol	150-76-3	0.37			
p-mentha-3,8-diene	586-67-4	6.50			
L-carvone	6485-40-1	0.30			
Camphene	79-92-5	0.31			
Cis-carveol	1197-06-4	1.70			
Cis-sabinol	3310-02-9	0.23			
Trans-p-mentha-1(7),8-dien-2-ol	374-16-7	3.04			
Dihydrocarveol	38049-26-2	0.39			
Neral	106-26-3	0.93			
Borneol	507-70-0	2.03			
Carvacrol	499-75-2	0.10			
Alpha-farnesene	26560-14-5	0.09			
Cis-p-mentha-1(7),8-dien-2-ol	374-16-8	0.40			
Carveol	1197-06-4	0.27			
**OTHER COMPOUNDS**					
Dimethyl sulfide	75-18-3	4.64	Ethyl caproate	123-66-0	0.92
Monoethylmalonate monoamide	7597-56-0	0.54	Ethyl heptanoate	106-30-9	0.79
1-Undecene	821-95-4	1.41	4-ethenyl-1,2-dimethyl-Benzene	27831-13-6	4.08
1-heptyl-2-methyl-Cyclopropane	74663-91-5	1.38	Verbenyl ethyl ether	80581-06-2	1.11
Lavandulyl acetate	498-16-8	0.21	Isopropyl methoxypyrazine	25773-40-4	0.84
Anethofuran	74410-10-9	0.23	4-ethyl-Phenol	123-07-9	4.83
1,2,3-trimethoxy-5-(2-propenyl)-Benzene	487-11-6	0.21	2,5-bis(1,1-dimethylethyl)-Phenol	5875-45-6	1.07
4-Ethylguaiacol	2785-89-9	3.55			
2-methoxy-Phenol	90-05-1	0.53			
2,4-Di-tert-butylphenol	96-76-4	0.43			
p-tert-butyl-Phenol	98-54-4	0.08			
4-ethyl-Phenol	123-07-9	9.84			
Isopropyl methoxypyrazine	25773-40-4	0.10			
7-(1-methylethylidene)-Bicyclo[4.1.0]heptane	53282-47-6	0.10			

Supplementary Table S2. Volatile organic compounds presented in sea fennel flower extracts detected by HS-SPME-GC/MS

Water extract			Hydroalcoholic extract		
**Name**	**CAS**	**% area**	**Compound**	**CAS**	**% area**
**ALCOHOLS**					
1-Hexanol	111-27-3	1.52			
1-Pentanol	71-41-0	0.21			
3-Octanol	589-98-0	0.08			
(E)-3-Hexen-1-ol	928-97-2	0.25			
1-Octanol	111-87-5	3.50			
2-ethyl-1-Hexanol	104-76-7	1.03			
Cis-p-2-menthen-1-ol	29803-82-5	0.32			
Trans-p-Mentha-2,8-dienol	139-65-3	0.66			
(4R,8R)-p-menth-1-ene-9-ol	13835-30-8	0.13			
Cis-p-mentha-1(7)8-dien-2-ol	374-16-8	0.45			
Trans-p-mentha-1(7)8-dien-2-ol	374-16-7	0.88			
p-Cymen-7-ol	536-60-7	0.10			
5-methyl-2-(1-methylethenyl)-4-Hexen-1-ol	58461-27-1	0.89			
Benzyl alcohol	100-51-6	0.22			
**TERPENOIDS**					
α - thujene	2867-05-2	1.29	α - thujene	2867-05-2	5.04
Camphene	79-92-5	0.07	Camphene	79-92-5	0.15
Cis-carveol	1197-06-4	0.87	α - pinene	80-56-8	0.13
Sabinene	3387-41-5	3.31	Sabinene	3387-41-5	7.85
2,4-thujadiene	36262-09-6	0.15	(+)-carvomenthene	1195-31-9	0.39
β myrcene	123-35-3	0.08	β myrcene	123-35-3	1.32
α Phellandrene	99-83-2	1.33	α Phellandrene	99-83-2	0.97
4-Carene	2950-33-7	4.32	4-Carene	2950-33-7	2.18
δ - 3 - carene	13466-78-9	6.35	δ - 3 - carene	13466-78-9	6.87
Spathulenol	6750-60-3	0.44	α-Terpinene	99-86-5	0.71
D-limonene	5989-27-5	1.45	D- limonene	5989-27-5	58.95
1,3,8-p-Menthatriene	18368-95-1	1.60	Ocimene	13877-91-3	1.01
P-Cymene	99-87-6	8.96	P-Cymene	99-87-6	12.26
Linalol	78-70-6	2.37	Valencene	4630-07-3	0.06
α - terpineol	10482-56-1	4.81	α - terpineol	98-55-5	0.17
4-terpinol	562-74-3	30.08	4-terpineol	562-74-3	0.49
Borneol	507-70-0	1.44	Caryophyllene	87-44-5	0.36
Carvacrol	499-75-2	0.20	Trans-Calamenene	73209-42-4	0.07
L-Carvone	6485-40-1	0.36	L-Carvone	6485-40-1	0.22
Thymol	89-83-8	0.14	Bornyl acetate	76-49-3	0.07

Sabinene hydrate	546-79-2	0.97	2,6-dimethyl- 2,4,6-Octatriene	7216-56-0	0.28
Trans-dihydrocarvone	5948-04-9	2.49			
para-mentha-3,8-diene	586-67-4	0.87			
Trans-Limonene oxide	4959-35-7	0.53			
**ALDEHYDES**					
2-methyl-Butanal	96-17-3	0.14			
α Campholenal	4501-58-0	0.30			
**KETONES**					
4-Isopropylcyclohexanone	5432-85-9	0.13			
α-irone	79-69-6	1.44			
2-heptanone	110-43-0	0.11			
Thujone	546-80-5	0.18			
α,β-Thujone	1125-12-8	0.20			
Sabina ketone	513-20-2	0.34			
Piperitone	89-81-6	0.08			
**OTHER COMPOUNDS**					
(E,Z) 2,4-Hexadiene	5194-50-3	0.39	Butylated Hydroxytoluene	128-37-0	0.15
3-ethenyl-1,2-dimethyl-1,4-cyclohexadiene	62338-57-2	1.95	1,6-dimethyl-4-(1-methylethyl)-naphthalene	483-78-3	0.04
Dimethyl sulfide	75-18-3	10.02	13-Hexyloxacyclotridec-10-en-2-one	127062-51-5	0.13
			2-Isopropyl-1-methoxy-4-methylbenzene	31574-44-4	0.13

Supplementary Table S3. Volatile organic compounds presented in rosemary extracts detected by HS-SPME-GC/MS

WATER EXTRACT			HYDROALCOHOLIC EXTRACT		
**Name**	**CAS**	**% Area**	**Name**	**CAS**	**% Area**
			**ALCOHOLS**		
1-Propanol	71-23-8	0.05			
2-methyl-1-propanol	78-83-1	0.09			
1-Hexanol	111-27-3	0.13			
Cis-3-hexenol	544-12-7	0.12			
3-Octanol	589-98-0	0.15			
6-methyl-5-Hepten-2-ol	1569-60-4	0.06			
2-ethyl-1-Hexanol	104-76-7	0.15			
Benzyl alcohol	100-51-6	0.04			
Phenethyl alcohol	60-12-8	0.03			
			**KETONES**		
Methyl Isobutyl ketone	108-10-1	0.07	3-Octanone	106-68-3	0.18
3-Hexanone	589-38-8	0.07	Verbenone	1196-01-6	0.48
4-methyl-3-Penten-2-one	141-79-7	0.59			
2-Heptanone	110-43-0	0.02			
3-Octanone	106-68-3	0.75			
6-methyl-5-Hepten-2-one	110-93-0	0.16			
Cis-isopinocamphone	15358-88-0	0.49			
Dihydrocarvone	7764-50-3	0.04			
Verbenone	1196-01-6	2.54			
			**TERPENOIDS**		
α - pinene	80-56-8	0.02	β- pinene	127-91-3	0.61
Camphene	79-92-5	0.10	α- pinene	80-56-8	19.91
β - myrcene	123-35-3	0.12	α -fenchene	471-84-1	0.26
4-carene	2950-33-7	0.03	Camphene	79-92-5	8.80
Eucalyptol	470-82-6	47.51	2,4-thjuadiene	36262-09-6	0.39
(Z)-beta-ocimene	3338-55-4	0.04	δ -3-carene	13466-78-9	0.07
P-Cymene	99-87-6	0.21	β- myrcene	123-35-3	8.38
Fenchone	1195-79-5	0.07	4-carene	2950-33-7	0.25
β-thjuone	471-15-8	0.02	D-limonene	5989-27-5	11.89
L - menthone	89-80-5	0.11	α -phellandrene	99-83-2	0.20
Linalol	78-70-6	2.24	Eucalyptol	470-82-6	17.85
Isopulegol	89-79-2	0.05	Trans- β -ocimene	3779-61-1	0.08
(±)-neoisopulegol	29141-10-4	0.10	P-Cymene	99-87-6	10.56
Fenchol	1632-73-1	0.36	Ylangene	14912-44-8	0.32
4-Terpineol	562-74-3	2.63	Cis-isopinocamphone	15358-88-0	0.06
Caryophyllene	87-44-5	0.03	4-Terpineol	562-74-3	0.55
L-Menthol	2216-51-5	0.35	Caryophyllene	87-44-5	2.48
α -terpineol	98-55-5	3.57	δ-terpineol	7299-42-5	0.09
Borneol	507-70-0	11.91	α- terpineol	98-55-5	0.97

Isopinocarveol	6712-79-4	0.58	Borneol	507-70-0	1.52
δ -terpineol	7299-42-5	0.66	α -gurjunene	489-40-7	0.15
D - carvone	2244-16-8	0.05	L-carvone	6485-40-1	0.17
Citronellol	106-22-9	0.06	δ -cadinene	483-76-1	0.13
Piperitone	89-81-6	0.03	α -Calacorene	21391-99-1	0.04
3-ethylidene-1-methyl-cyclopentene	62338-00-5	0.07	α - Elemol	639-99-6	0.05
(Z)-sabinol	3310-02-9	0.05	13-Hexyloxacyclotridec-10-en-2-one	127062-51-5	0.08
cis-carveol	1197-06-4	0.06	Ethyl linalol	10339-55-6	0.33
3-caren-5-one	81800-50-2	0.02	Camphor	464-49-3	5.16
Methyl eugenol	93-15-2	0.05	Humulene	6753-98-6	0.41
Thymol	89-83-8	0.09	Trans-calamenene	73209-42-4	0.20
Eugenol	97-53-0	0.09			
Carvacrol	499-75-2	0.06			
Camphor	464-49-3	22.47			
**OTHER COMPOUNDS**					
Toluene	108-88-3	0.03	2,4-dimethyl-heptane	2213-23-2	0.14
2,4-thujadiene	36262-09-6	0.08	Tridecane	629-50-5	0.78
Bornyl acetate	76-49-3	0.44	Tetradecane	629-59-4	0.03
1,6-dimethyl-4-(1-methylethyl)-naphthalene	483-78-3	0.01	Copaene	3856-25-5	0.82
			Bornyl acetate	76-49-3	5.37
2,5-bis(1,1-dimethylethyl)-phenol	5875-45-6	0.04	β-Amorphene	483-75-0	0.15
			Cadalene	483-78-3	0.03
13-Hexyloxacyclotridec-10-en-2-one	127062-51-5	0.05	1,2,3,6-tetramethyl-Bicyclo[2.2.2]oct-2-ene	62376-14-1	0.07

Supplementary Table S4. Volatile organic compounds presented in olive leaf extracts detected by HS-SPME-GC/MS

WATER EXTRACT			HYDROALCOHOLIC EXTRACT		
**Name**	**CAS**	**% Area**	**Name**	**CAS**	**% Area**
		**ALCOHOLS**			
2-methyl-1-Propanol	78-83-1	4.68	Benzyl alcohol	100-51-6	11.74
3-methyl-1-Butanol	123-51-3	12.03	Phenethyl alcohol	60-12-8	26.21
3-methyl-3-Buten-1-ol	763-32-6	0.49			
2-Heptanol	543-49-7	0.29			
(E)-4-Hexen-1-ol	928-92-7	0.39		**KETONES**	
6-methyl-5-hepten-2-ol	1569-60-4	1.59	2-Nonanone	821-55-6	15.77
2-ethyl-1-hexanol	104-76-7	1.19	3-methyl-Nonane	5911-04-6	3.94
Phenethyl alcohol	60-12-8	8.53			
		**TERPENOIDS**			
Eucalyptol	470-82-6	0.73	Terpinen-4-ol	562-74-3	12.78
P-cymene	99-87-6	0.08	Alpha terpineol	10482-56-1	8.18
(±)-Theaspirane	36431-72-8	54.74	Anethol	104-46-1	5.68
Linalool	78-70-6	0.82			
Terpinen-4-ol	562-74-3	1.10			
α- terpineol	10482-56-1	1.49			
Di-hydro-beta-ionol	3293-47-8	7.69			
β-Bisabolol	15352-77-9	0.41			
Drim-7-en-11-ol	468-68-8	0.41			
		**OTHER COMPOUNDS**			
2-methylfuran	534-22-5	2.71	2,4,6-trimethyloctane	62016-37-9	15.69
2,4-Di-tert-butylphenol	96-76-4	0.62			

Supplementary Table S5. Volatile organic compounds presented in mock privet leaf extracts detected by HS-SPME-GC/MS

WATER EXTRACT			HYDROALCOHOLIC EXTRACT		
**Name**	**CAS**	**% Area**	**Name**	**CAS**	**% Area**
**ALCOHOLS**					
1-Pentanol	71-41-0	0.72	(Z)-3-Hexen-1-ol formate	33467-73-1	7.45
2-Heptanol	543-49-7	2.13			
1-Hexanol	111-27-3	2.10	(E)-3-Hexen-1-ol	928-97-2	5.29
3-Hexenol	544-12-7	0.86	Benzyl alcohol	100-51-6	9.11
2-Octanol	123-96-6	3.06			
2-Ethyl-1-hexanol	411-44-8	2.95			
2-Nonanol	628-99-9	0.83			
Benzyl alcohol	100-51-6	0.24			
Phenethyl alcohol	60-12-8	0.54			
**KETONES**			**ALDEHYDES**		
2-Heptanone	110-43-0	10.38	Hexanal	66-25-1	6.45
3-Octanone	106-68-3	0.23			
2-Octanone	111-13-7	1.71			
Verbenone	1196-01-6	0.28			
**TERPENOIDS**					
Eucalyptol	470-82-6	53.31	Linalol	78-70-6	32.58
P-Cymene	99-87-6	0.65	α -terpinol	98-55-5	9.37
Linalol	78-70-6	1.69	D-Limonene	5989-27-5	1.55
L-menthol	2216-51-5	0.39	Anethol	104-46-1	4.07
α -terpinol	98-55-5	1.12	β-Cyclocitral	821-55-6	9.39
Borneol	507-70-0	4.72	Damascenone	23696-85-7	11.71
Eugenol	97-53-0	0.18	β-Cyclocitral	432-25-7	3.04
Calamenene	483-77-2	0.23			
D-carvone	2244-16-8	0.12			
Camphor	464-49-3	9.24			
**OTHER COMPOUNDS**					
3-methylfuran	930-27-8	0.47			
2,4-Di-tert-butylphenol	96-76-4	0.82			
2-Methylpropanoic acid	77-68-9	0.61			
Methoxy-phenyl-oxime	222-86-6	0.42			

Supplementary Table S6. Volatile organic compounds presented in lemon balm leaf extracts detected by HS-SPME-GC/MS

WATER EXTRACT			HYDROALCOHOLIC EXTRACT		
**Name**	**CAS**	**% Area**	**Name**	**CAS**	**% Area**
**ALCOHOLS**					
2-methyl-1-Propanol	78-83-1	0.65	2,4-Di-tert-butylphenol	96-76-4	8.61
Phenethyl alcohol	60-12-8	0.45	2-ethyl-1-Hexanol	104-76-7	11.09
6-methyl-5-Hepten-2-ol	1569-60-4	0.79			
2-ethyl-1-Hexanol	104-76-7	3.23			
1-octen-3-ol	3391-86-4	5.75			
Cis-3-hexenol	544-12-7	4.01			
**KETONES**			**ALDEHYDES**		
1-heptyl-2-methyl-Cyclopropane	74663-91-5	1.01	Benzaldehyde	100-52-7	17.54
3-Octanone	106-68-3	3.15			
5,6,7,7a-tetrahydro-4,4,7a-trimethyl-, (R)-2(4H)-Benzofuranone	17092-92-1	0.38			
Acetoine	513-86-0	8.70			
**TERPENOIDS**					
α -terpineol	98-55-5	0.83	D-limonene	5989-27-5	8.54
Linalol	78-70-6	6.73	Rose oxyde	16409-43-1	4.48
Isoeugenol	97-54-1	0.71	L-menthol	2216-51-5	27.13
Thymol	89-83-8	0.42	D-carvone	2244-16-8	7.83
Citronellol	106-22-9	8.15	Citronellol	106-22-9	5.94
4-terpineol	562-74-3	0.68			
Geraniol	106-24-1	1.167			
Eucalyptol	470-82-6	3.67			
L-menthol	2216-51-5	9.59			
Eugenol	97-53-0	0.75			
2,5,6-trimethyl-1,3,6-Heptatriene	42123-66-0	0.39			
Pulegone	89-82-7	0.34			
5-methyl-2-(1-methylethyl)-(1.alpha.,2.beta.,5.beta.)-Cyclohexanol	490-99-3	2.94			
L-menthone	89-80-5	1.13			
Rose oxyde	16409-43-1	0.54			
P-cymene	99-87-6	0.71			
**OTHER COMPOUNDS**					
Propanoic acid, ethyl ester	105-37-3	0.71	Phenol, 3,5-bis(1,1-dimethylethyl)-	1138-52-9	8.85
Isoamyl acetate	123-92-2	1.15			

Methyl Salicyate	119-36-8	1.39
3-methyl-Furan	930-27-8	2.06
Dimethyl sulfide	75-18-3	16.75
2,5-diethyltetrahydro-Furan	41239-48-9	0.48
2,4-Di-tert-butylphenol	96-76-4	0.96
